# Defect-Rich CuZn Nanoparticles for Model Catalysis Produced by Femtosecond Laser Ablation

**DOI:** 10.1021/acsami.4c07766

**Published:** 2024-06-27

**Authors:** Niusha Lasemi, Thomas Wicht, Johannes Bernardi, Gerhard Liedl, Günther Rupprechter

**Affiliations:** † Institute of Materials Chemistry, TU Wien, 1060 Wien, Austria; ‡ University Service Center for Transmission Electron Microscopy, TU Wien, 1020 Wien, Austria; § Institute of Production Engineering and Photonic Technologies, TU Wien, 1060 Wien, Austria

**Keywords:** ultrashort pulses, CuZn nanoalloys, stacking fault, dislocation, twinning, ethylene hydrogenation

## Abstract

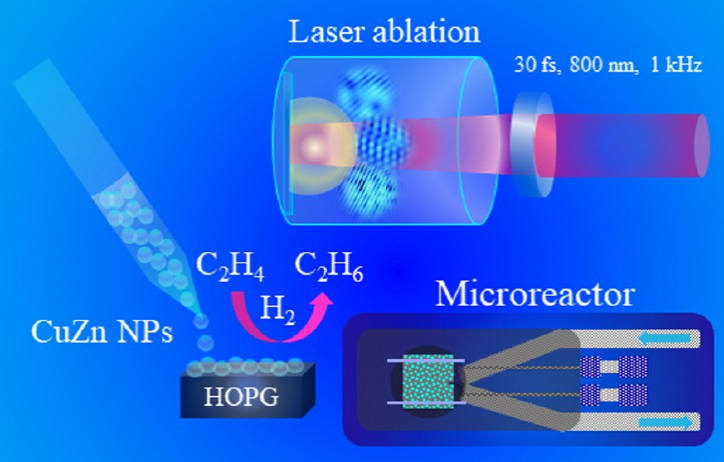

Femtosecond laser ablation of Cu_0.70_Zn_0.30_ targets in ethanol led to the formation of periodic surface nanostructures and crystalline CuZn alloy nanoparticles with defects, low-coordinated surface sites, and, controlled by the applied laser fluence, different sizes and elemental composition. The Cu/Zn ratio of the nanoparticles was determined by energy dispersive X-ray spectroscopy, X-ray photoelectron spectroscopy, and selected area electron diffraction. The CuZn nanoparticles were about 2–3 nm in size, and Cu-rich, varying between 70 and 95%. Increasing the laser fluence from 1.6 to 3.2 J cm^–2^ yielded larger particles, more stacking fault defects, and repeated nanotwinning, as evident from high-resolution transmission electron microscopy, aided by (inverse) fast Fourier transform analysis. This is due to the higher plasma temperature, leading to increased random collisions/diffusion of primary nanoparticles and their incomplete ordering due to immediate solidification typical of ultrashort pulses. The femtosecond laser-synthesized often nanotwinned CuZn nanoparticles were supported on highly oriented pyrolytic graphite and applied for ethylene hydrogenation, demonstrating their promising potential as model catalysts. Nanoparticles produced at 3.2 J cm^–2^ exhibited lower catalytic activity than those made at 2.7 J cm^–2^. Presumably, agglomeration/aggregation of especially 2–3 nm sized nanoparticles, as observed by postreaction analysis, resulted in a decrease in the surface area to volume ratio and thus in the number of low-coordinated active sites.

## Introduction

Pulsed laser ablation in liquid (PLAL) is an innovative technique to produce nanomaterials, also considered “green” as only metal/alloy/oxide targets and nontoxic liquids are used. Steering the process requires considering several factors, including the physicochemical properties of the target, the laser parameters, and the ablation environment. The application of PLAL-derived nanoparticles (NPs) has already wide applications, but is rather new in catalysis.^1−3^ Recently, Müller and co-workers^2^ highlighted the application of lasers for tailored synthesis of catalytic nanomaterials.

Heterogeneous catalysis contributes to at least 80% of all produced chemicals. Thus, developing and improving methods to synthesize highly efficient catalysts is important. In heterogeneous catalysis, a chemical reaction occurs at an interface (e.g., solid/gas) and several processes including adsorption/desorption, diffusion, and reaction take place.^4−9^ These processes are governed by the physicochemical properties of the catalyst, which, in turn, are determined by its atomic structure and composition. Transition metals, as well as their oxides, are effective catalysts for various applications, also benefiting from their low cost as compared to precious metals such as Pt, Pd, Rh, or Ir.

Apart from (ordered) crystal structures, so-called defect engineering has become a strategy to improve catalytic activity.^10−15^ Undoubtedly, the abundance, distribution, and nature of defect structures affect catalytic performance. Understanding this interaction calls for precise studies of defect chemistry and how to control the synthesis of defect-rich heterogeneous catalysts. Many examples can be found in the literature, but how such defects exactly affect the reaction is still under debate. Catalysts based on Cu and Cu-oxide have been extensively used for many reactions, due to their favorable activity and selectivity, such as in (preferential) CO oxidation^16−19^ or CO_2_ hydrogenation.^12,20−24^

Strained Cu NPs^20^ in Cu/ZnO/Al_2_O_3_ catalyst exhibited higher activity in methanol synthesis, pointing to the role of lattice strain and defects in the enhancement of catalytic activity. Based on studies of active sites in Cu/ZnO/Al_2_O_3_ for methanol synthesis,^25^ the high activity relies on the existence of stacking faults (SF) in Cu NPs. SF defects (a type of planar defect) and line defects can create steps and kinks at the nanoparticle surface, all of which are beneficial for catalytic performance. Also, the modification of Cu steps by Zn species (the nature of which is debated and may vary) are further reasons for active catalysts.^21−26^

It was also reported that methanol synthesis takes place at step-edge sites of copper surfaces, with Cu NPs >8 nm being the most active.^21^ Furthermore, the Cu/ZnO activity is ∼3–5 times higher than that of catalysts without ZnO.^21^ Also, wetting of ZnO by Cu caused dynamical changes in Cu morphology.^26^ For CuZn model catalysts,^22^ surface oxidation under reaction conditions and formation of ZnO/Cu was observed. Accordingly, Cu/ZnO interfaces or CuZn ensembles are essential for creating certain active sites that are, however, still debated. For bimetallic CuZn NPs on various supports, the dynamic nature of active sites was studied by operando X-ray absorption and photoelectron spectroscopy, revealing Zn surface segregation and formation of a ZnO-rich shell on CuZn/SiO_2_.^24^ In general, bimetallic catalyst may exhibit properties exceeding by far those of the individual metals.^27−31^

When using PLAL, its environment (i.e., high-temperature plasma and cavitation bubble) naturally produces nanoparticles with defects, low-coordinated surface sites, and lattice strain, which can be quite active. However, only a few studies exist so far on laser generation of bimetallic CuZn NPs^32−36^ in liquids. There is even only one report of applying PLAL-synthesized CuZn in electrocatalysis: nanosecond laser ablation of a CuZn target in water resulted in CuZnO_*x*_ NPs that were used for selective electrochemical reduction of CO_2_, displaying good selectivity toward C_2_H_4_ due to a claimed synergistic interaction between Cu and Zn.^36^ Besides the bimetallic composition, the structure of defect-rich Cu NPs should also affect the catalytic performance.^25^

It is also well established for metal nanoparticles that their atomic structure (lattice contraction/expansion), shape (facets), and electronic properties are typically size-dependent.^5,8,9^ Smaller particles typically expose more open planes (low-coordinated sites) than larger particles^37,38^ and this can be well controlled by wet-chemical synthesis methods. However, defect-rich nanostructures cannot be easily obtained and maintained. Accordingly, to produce defect-rich CuZn nanoparticles, which cannot be easily achieved by conventional chemical methods, femtosecond (fs) laser ablation was applied herein. This has two general advantages: (i) Ultrashort interaction with (thermally conductive) metals results in a negligible heat-affected zone, since the pulse duration is shorter than the electron–lattice relaxation time; thus, laser energy is not wasted as heat, which increases NP productivity. (ii) Based on theoretical studies, the effective cooling rate in the femtosecond regimes is 10^12^ K s^1–^,^39^ which is the fastest among other longer pulse durations. PLAL is thus a useful method to produce ligand-free nanoparticles with intrinsic defects in their crystal structure.^2,3^ This strategy had been successfully applied by laser ablation of a Ag target, which produced highly strained Ag nanoparticles (with stacking faults) that were very active for hydrogen generation.^40^ Note that the PLAL approach is inherently different from modifying chemically synthesized nanostructures by subsequent exposure to low fluence femtosecond laser pulses.^41^

CuZn PLAL nanoparticles could also be used for other applications, such as composites (with improved hydrophobic, mechanical, and conductive properties),^42^ environmentally friendly, nontoxic antifungal^43^ or antimicrobial agents for wastewater treatment,^44^ or to combat multidrug-resistant bacteria.^45^

In the current work, femtosecond laser irradiation of a CuZn solid surface was employed for CuZn nanoparticle formation in ethanol at various fluences, with the CuZn targets characterized by various techniques including optical microscopy, profilometry, scanning electron microscopy (SEM), energy dispersive X-ray spectroscopy (EDX), micrograzing incidence X-ray diffraction (micro-GIXRD), micro-Raman spectroscopy, and X-ray photoelectron spectroscopy (XPS). The produced alloy nanoparticles were analyzed by micro-Raman spectroscopy, ultraviolet/visible (UV/vis) spectroscopy, dynamic light scattering (DLS), XPS and electron microscopy techniques, including transmission electron microscopy (TEM), high-resolution transmission electron microscopy (HRTEM), selected area diffraction (SAED), and EDX mapping. The produced CuZn NPs were indeed defect-rich and were subsequently supported on highly oriented pyrolytic graphite before being tested for gas phase ethylene hydrogenation. In addition, CuZn nanoparticles produced at 1.6, 2.1, 2.7, and 3.2 J cm^–2^ were supported on carbon-coated TEM-grids and exposed to the same reaction conditions, enabling postreaction analysis.

## 2 Materials and Methods

### 2.1 Material

A commercial alloy target, CuZn (brass) foil with a thickness of 0.6 mm was used to produce bimetallic nanoparticles via PLAL. The polycrystalline CuZn target containing 70 atom % Cu and 30 atom % Zn was purchased from Thermo Scientific company. Pure solvent (ethanol, p.a. 99.5%) was purchased from Sigma-Aldrich. In addition, the ethanol solvent was purified by a polypropene filter (pore size of 0.45 μm, VWR).

### 2.2 Grinding and Polishing

CuZn targets (1 × 1 cm^2^) were treated by SiC grinding papers (Grit 1200, 2400, and 4000) inside an ALLIED grinding device. All targets were also polished by magnetic MD-Mol disks of 1 and 3 μm. For final polishing, targets were transferred to the VibroMet (BUEHLER) by applying 10% vibration for 48 h inside nondrying fumed silica suspension to achieve 0.04 μm of grain size. Last, all targets were cleaned ultrasonically (LAVAMIN), with ultrasound waves combined with water rinsing.

### 2.3 Femtosecond Laser Arrangement

**Figure 1:**
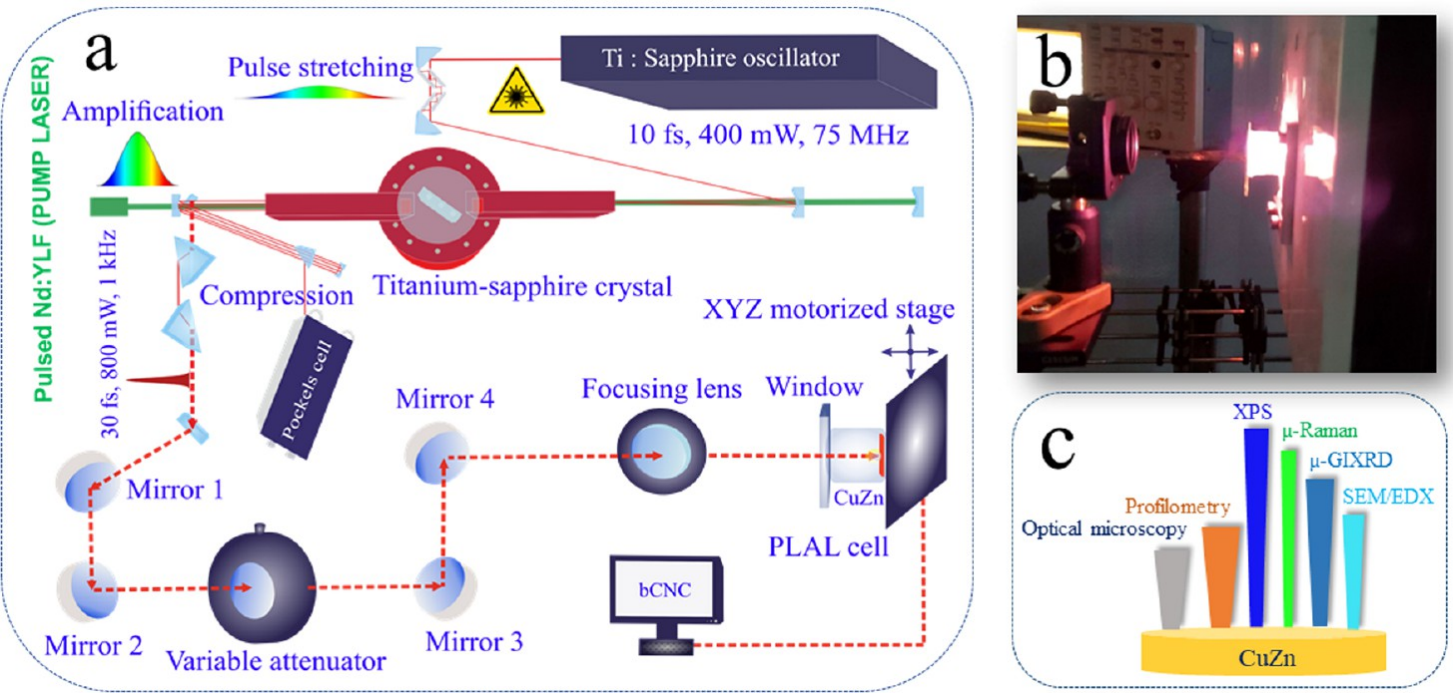
(a) Schematic of the femtosecond laser setup showing the main parts: oscillator, amplification area, and pump source. The PLAL cell with the target (orange) was positioned on a motorized XYZ scanning stage; (b) photograph shows an ongoing ablation process for nanoparticle synthesis in liquid; (c) overview of analytical techniques applied to characterize the CuZn target including optical microscopy, profilometry, X-ray photoelectron spectroscopy, confocal micro-Raman spectroscopy, micrograzing incidence X-ray diffraction, and scanning electron microscopy in conjunction with energy dispersive X-ray spectroscopy mapping.

The experimental PLAL setup is described in detail below (Figure 1). A CuZn target was fixed at the bottom of a glassy PLAL cell with a thickness of 15 mm, and with its window positioned perpendicularly toward the horizontal laser beam. The PLAL cell was mounted on a motorized XYZ stage. The fs-laser includes a seed laser, i.e., a commercial femtosecond system (SPECTRA-PHYSICS, *P* ≤ 400 mW, 800 nm, 10 fs, 75 MHz) and a pump laser, i.e., a continuous Nd/YLF laser at 532 nm.^46^

A so-called chirped pulse amplification (CPA) was done by pairs of third-order dispersion precompensating mirrors to stretch the pulse before amplification. After the laser beam was directed to the amplification area (Ti-sapphire crystal) and the system was pumped with a green laser, a pair of prisms was used to compress the final pulse. The final output was *P* ≤ 1 W, 30 fs, 1 kHz.

To adjust the focal point on the CuZn target in ethanol, a fused silica Plano-convex lens (FS lens, EKSMA OPTICS) with a diameter of 25.4 mm and a focal length of 100 mm was used. The target was ablated by 100 mW output power and 1000 shots (*N*_1000_) for different distances from the lens.

The femtosecond laser system was equipped with a computer-controlled motorized scanning stage to automatically move the PLAL cell with the target in order to ablate fresh target areas after 1000 pulses (repeated about 300 times for NP production). To adjust the output ablation power, a variable attenuator, including a beam splitter and a polarizer with a quartz half-wave plate, was used. The output power was controlled by a power meter (OPHIR Photonics). The squared diameter of the ablated area (*D*^2^) on CuZn at various *F* was evaluated by using optical microscopy (Zeiss AxioVision software). For this evaluation (Figure S1), an average of 30 craters was considered. The calculated fluences ranged from 1.0 to 3.2 J cm^–2^ and are summarized in Table T1.

All characterization techniques, used to study the targets and colloidal nanoparticles, are thoroughly described in the Supporting Information: scanning electron microscopy (Supporting Note 2.1), profilometry (Supporting Note 2.2), micrograzing incidence X-ray diffraction (μ-GIXRD) (Supporting Note 2.3), dynamic light scattering (DLS) (Supporting Note 2.4), confocal micro-Raman spectroscopy (Supporting Note 2.5), UV/vis spectroscopy (Supporting Note 2.6), transmission electron microscopy (Supporting Note 2.7), and X-ray photoelectron spectroscopy (Supporting Note 2.8).

### 2.4 Catalytic Tests

For catalytic tests, a flow microreactor setup was used that is particularly well suited for model catalysts, as described in.^47,48^ Mass flow controllers (MFCs) were used to adjust the gas flow to the reaction cell, employing high-purity (5.0) gases supplied by Messer Austria. A differentially pumped mass spectrometer (Hiden HPR 20) and a gas chromatograph (Micro GC Fusion, INFICON) were used to analyze the feed composition at the reactor outlet.

## 3 Results and Discussion

As mentioned above, PLAL can generate high Miller index nanoparticles with low-coordinated surface sites, crystal defects (e.g., stacking faults, dislocations, twin boundaries), and internal strain, all creating active centers. By varying the laser fluence, the size (distribution) of NPs and, in case of alloys, their composition can be modified.^2,3,49^ Nevertheless, it is quite challenging to obtain a uniform composition. Below, we describe the femtosecond ablation of CuZn targets and NP analysis in solution. This is followed by a thorough characterization of the produced defect-rich CuZn nanoparticles. Finally, the defect-rich CuZn NPs were supported on a HOPG and tested in catalytic ethylene hydrogenation in an atmospheric pressure flow reactor. In addition, postreaction analysis was applied to study the CuZn NPs′ stability under reaction conditions.

### 3.1 Analysis of the Pristine CuZn Target

**Figure 2:**
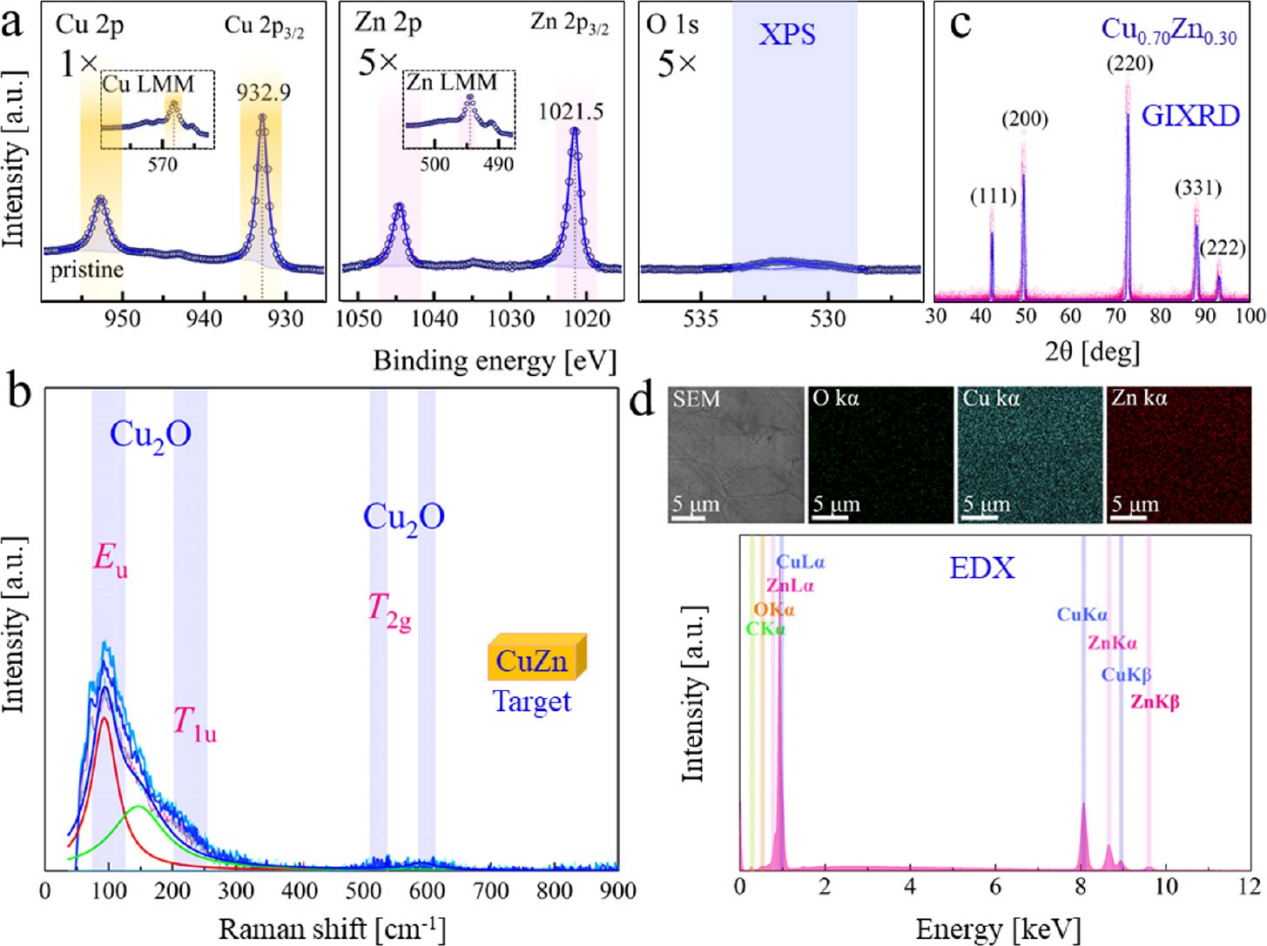
Analysis of the pristine (polished) CuZn target. (a) XPS spectra of Cu 2p, Zn 2p, and O 1s; the envelope of the fit is shown as a blue solid line; (b) μ-Raman spectrum of active and inactive modes; a Lorentz fit was applied; cumulative fits and corresponding deconvoluted peaks are applied based on the best *R*^2^: 0.95; (c) μ-GIXRD pattern; a Gauss fit was applied; cumulative fit is applied based on the best *R*^2^: 0.96; (d) SEM and elemental EDX images, and EDX spectrum of the polished CuZn alloy.

To confirm the elemental composition in the commercial Cu_0.70_Zn_0.30_ alloy and to provide a solid reference for postablation characterization, several techniques were applied for pristine target analysis. The Cu/Zn ratio (at.%) obtained by XPS, μ-GIXRD, and EDX were 83:17, 70:30, and 72:27, respectively, with XPS suggesting some Cu surface segregation. XPS spectra of the pristine (polished) CuZn foil are presented in Figure 2a. The Cu 2p region was fitted with a spin–orbit splitting of 19.8 eV between Cu 2p_3/2_ and Cu 2p_1/2_ and a peak area ratio of 2:1. The Cu 2p_3/2_ peak is located at 932.7 eV, in line with either Cu or Cu_2_O. The Cu LMM region (inset) revealed a peak at 568.0 eV (*h*ν: 1486.6 eV, kinetic energy: 918.6 eV). The modified Auger parameter (α)′ value of 1851.3 eV matches well with that of metallic Cu. The Zn 2p region showed a doublet separated by 23.0 eV with Zn 2p_3/2_ located at 1021.5 eV. The α′ value of 2013.6 eV is in good agreement with literature values for metallic Zn.^50,51^ In the O 1s region, only ∼6 at. % of oxygen was detected, with only ∼1:3 corresponding to metal oxides (lowest BE peak at 530.2 eV).

Similar to XPS, minor surface oxidation was observed by micro-Raman spectroscopy (Figure 2b), which may be related to a native oxide layer, indicating vibrational modes of Cu_2_O including 2-fold (E_u_) and 3-fold (T_1u_ and T_2g_) degenerate symmetries. Cu_2_O showed an intense peak at 89–93 cm^–1^ (inactive Raman mode; E_u_) and double peaks at ∼529–533 and 621 cm^–1^. Based on group theory analysis, the Raman spectrum of a perfect Cu_2_O crystal should exhibit only the T_2g_ mode at ∼515 cm^–1^ (Raman active), but different one-phonon scatterings are responsible for detecting inactive Raman modes.^52^ A single peak at ∼621 cm^–1^ may also have been an indication of CuO formation, which could overlap with the Cu_2_O doublet. Since no doublet was detected from ∼300 to 350 cm^–1^, CuO may not be formed, like the XPS result, with no satellites of CuO detected. ZnO peaks (250 to 500 cm^–1^) were also not observed.

Diffraction patterns of CuZn targets were obtained by micro-GIXRD (Figure 2c), with the peaks corresponding to JCPDS card no. 04–006–2621. SEM/EDX mapping of the pristine target (Figure 2d) showed a homogeneous distribution of Cu and Zn with only slight oxidation, in line with XPS, Raman, and GIXRD.

Laser ablation produced craters with a maximum depth of ∼1 μm (Figure S2), paralleled by the generation of laser-induced periodic surface structures (LIPSS). Based on our previous study of LIPSS on NiAu,^53^ LIPSS is the stage preceding generation of nanoparticles in liquids. Scanning electron microscopy (Figures S3 and S4) showed the formation of self-organized surface structures at the ablated areas with a periodicity of low spatial frequency and deviation angles between ∼5.3 and 8.6°. The deviation angle was increased by increasing *F* due to higher temperature, liquid evaporation, and consequent Marangoni bursting.^53,54^ To ensure that nanoparticle production is initiated in liquid and to avoid plasma confinement/reshaping at deep cavities, the applied laser fluence should be equal to the threshold fluence for nanoripple formation. A full analysis and discussion of CuZn craters and LIPSS is presented in the Supporting Information (Figures S1–S7 and Tables T1–T4).

### 3.2 CuZn Nanoparticle Analysis

Bimetallic CuZn nanoparticles produced at fluences of 1.6 to 3.2 J cm^–2^ in ethanol were characterized by DLS to determine their hydrodynamic properties. Colloidal NPs were also analyzed by micro-Raman spectroscopy and UV/vis spectroscopy to study their chemical composition and optical properties, respectively. TEM/HRTEM/SAED/HAADF/EDX of CuZn nanoparticles were applied to study their shape, composition, crystallinity, defect structures, and elemental distribution. For further analysis regarding the chemical composition, XPS analysis was performed for CuZn nanoparticles deposited on a HOPG.

#### 3.2.1 Analysis of Colloidal CuZn NPs

Micro-Raman spectra of colloidal CuZn nanoparticles are shown in Figure S8. Only Raman peaks of ethanol were detected (signals of Cu_2_O (T_2g_ mode at ∼515 cm^–1^) or ZnO were absent), indicating that the CuZn NPs were metallic and contaminations were absent.

**Figure 3:**
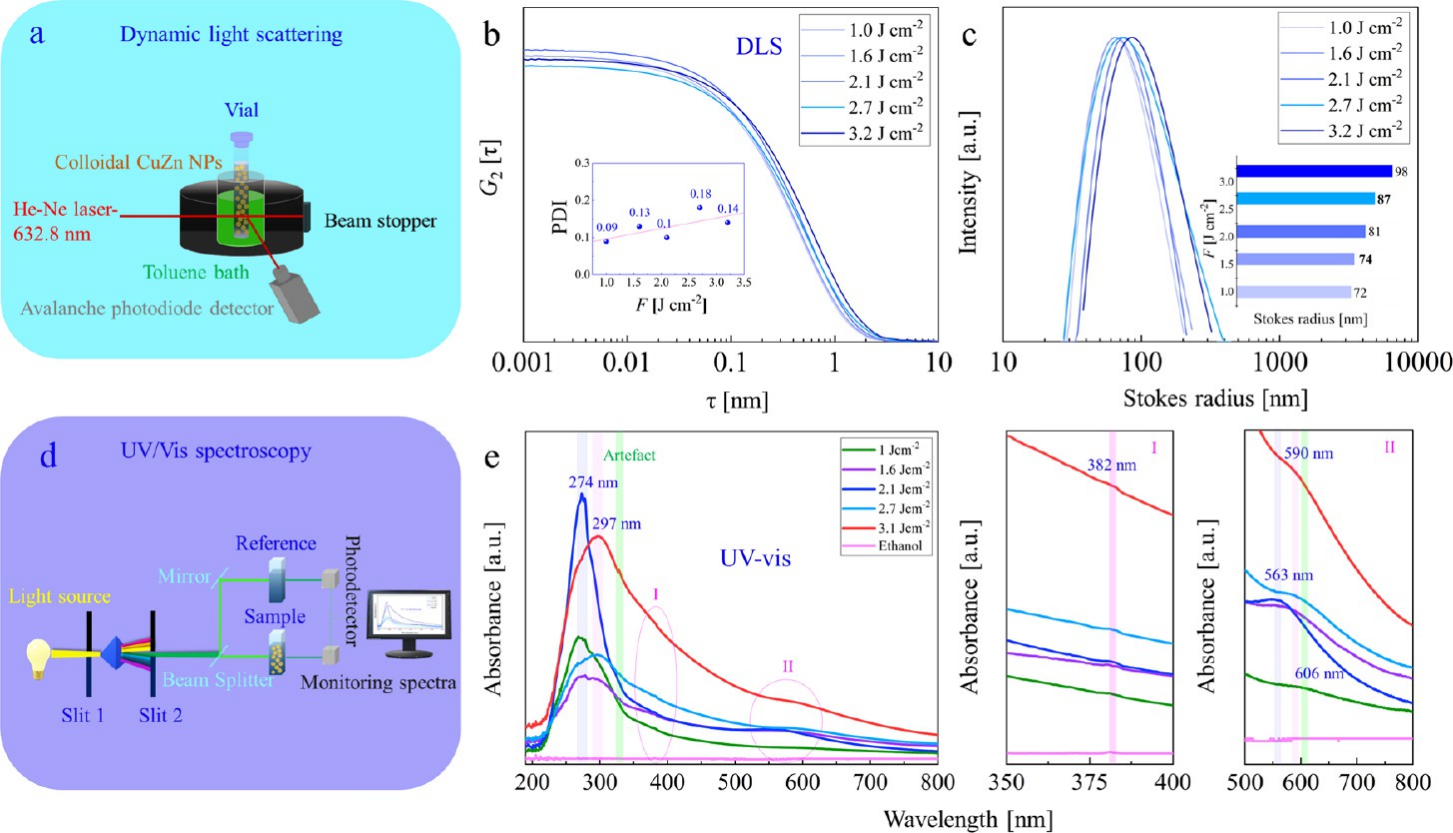
Analysis of colloidal CuZn nanoparticles synthesized by fs-PLAL at various *F*. (a) Schematic of DLS analysis; (b) autocorrelation function of intensity versus delay time (τ), inset shows PDI versus *F* (Cumulant Fit); (c) Stokes radius distribution with inset displays Stokes radii [nm] for various applied fluences *F*; (d) schematic of UV/vis spectroscopy; (e) UV/vis absorption spectra and magnified regions I (Zn) and II (Cu).

Dynamic light scattering (DLS) (indicating the hydrodynamic characteristics of the CuZn nanofluids) and UV/vis results are shown in Figure 3. Schemes illustrating the analysis of colloidal CuZn NPs by DLS and UV/vis spectroscopy are displayed in Figure 3a,d, respectively.

The PLAL process allows control of the nanoparticle size distribution via the applied laser fluence. Previous studies on the effect of laser fluence showed that the polydispersity and size increased upon increasing the laser fluence.^3,49,55^ Higher laser fluence results in the formation of a high-pressure and high-temperature plasma, higher temperature in the bubble, and subsequently an increased Brownian motion, which increases the NP collision probability and thus the particle size. All autocorrelation curves (Figure 3b), *G*_2_(τ) versus delay time (τ), show smooth decays, thus no sedimentation or colloidal instability occurred during measurements. The lowest polydispersity of well-defined CuZn NPs was observed at *F*: 1 J cm^–2^ and a narrower size distribution was achieved.^46,49^

The Stokes radius of the CuZn NPs increased upon increasing *F* (Figure 3c). DLS shows larger mean sizes for 2.7 and 3.2 J cm^–2^ than for lower *F*, which are similar. At higher *F*, higher nanoparticle productivity was obtained, but at the expense of narrow size distribution (due to particle–particle diffusion/coalescence or Ostwald ripening^3,49^). Obviously, in a colloidal system, smaller NPs can diffuse faster, so their *D*_trasn_ and *D*_rot_ coefficients are bigger than those of larger NPs (Table T5).

Note that DLS showed larger average sizes (Table T5) than the mean particle sizes determined by TEM (see below). Although DLS is a useful technique for analyzing the average size of colloidal NPs, it typically overestimates the actual values, as shown in previous studies of Si^49^ and NiAu^56^ nanoparticles, femtosecond-produced in liquids. This is very likely due to agglomeration, which, together with the solvation shell, can mimic larger particle sizes (the light scattering intensity is proportional to the particle diameter to the power of six). Still, DLS shows a trend in size evolution.

UV/vis spectroscopy (Figure 3e) displayed double peaks at (∼274–297 nm) and (∼563–606 nm), which are characteristic absorption peaks of Cu.^57^ Due to the increase in NP size upon increasing *F*, the intense peak of Cu at ∼274 nm for fs-PLAL NPs produced at 1, 1.6, and 2.1 J cm^–2^ red-shifted to ∼297 nm for the 2.7 and 3.2 J cm^–2^ samples. Furthermore, the absorption peak of the 3.2 J cm^–2^ sample is broad, confirming the abundance of larger NP sizes. An intense peak in the 2.1 J cm^–2^ sample seems related to the higher abundance of small NP sizes (2–3 nm) when compared with the second mode (20–50 nm) detected by TEM (see below). A low intensity peak of Cu (Figure 3e II) appeared regardless of NP size and could be due to a different Cu/Zn ratio. A very small peak at 382 nm (Figure 3e I) corresponds to Zn absorption, which would be more intense for ZnO,^58^ confirming the absence of ZnO as indicated by Raman.

#### 3.2.2 Electron Microscopy of CuZn NPs

To study the shape, crystal structure, defects, and elemental distribution of CuZn nanoalloy particles, several electron microscopy techniques were applied to the samples prepared using *F* of 1.6, 2.1, 2.7, and 3.2 J cm^–2^. Based on previous studies, such fluences avoid unwanted nonlinear processes.^49,56,59^

**Figure 4:**
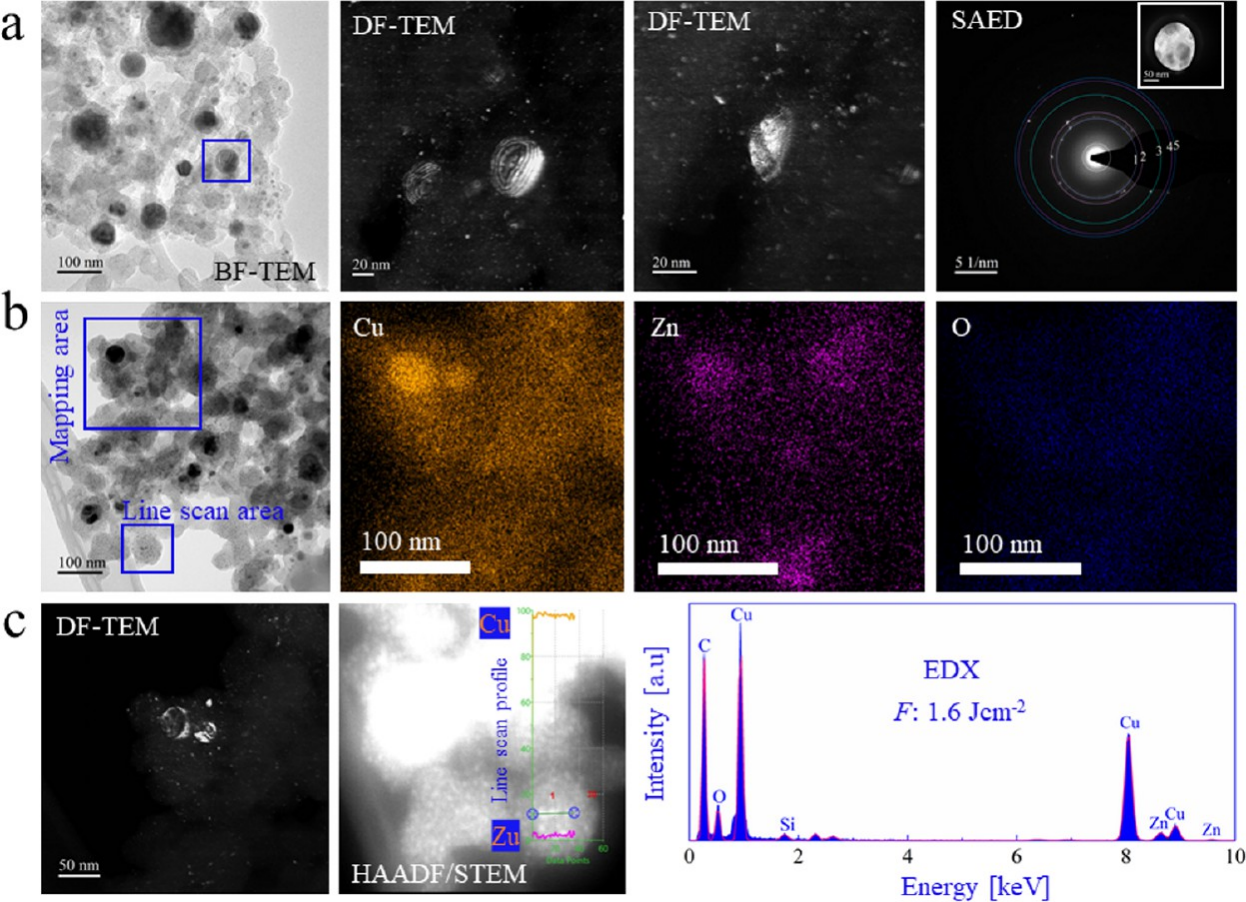
Transmission electron microscopy and EDX analysis of CuZn nanoparticles synthesized by fs-PLAL at *F*: 1.6 J cm^–2^. (a) BF-TEM, DF-TEM (showing thickness fringes), and SAED using an aperture selecting the area of interest; (b) BF-TEM of another area with blue squares marking the EDX mapping of Cu, Zn, and O and the region of the line scan in panel (c); (c) DF-TEM and HAADF-STEM of a small CuZn nanoparticle with EDX line scan overlay and EDX spectrum.

BF-TEM micrographs of CuZn nanoalloys (Figure 4a) synthesized at 1.6 J cm^–2^ revealed a bimodal size distribution. Larger NPs had a mean size of 38 nm, the small ones were 2 nm (for a size distribution analysis, see Figure S11ab). DF-TEM displayed a series of thickness fringes for larger CuZn nanoparticles, illustrating a three-dimensional (3D) near-spherical shape.^60^ SAED patterns (Figure 4) were acquired to determine the Cu/Zn ratio, with reference lattice distances and experimental results summarized in Tables T6 and T7, correspondingly. The observed interplanar spacings indicate Cu-rich alloys, but they are all quite similar, preventing an exact assignment (Cu_0.70_Zn_0.30_, Cu_0.75_Zn_0.25_, Cu_0.85_Zn_0.15_, and Cu_0.95_Zn_0.05_). Moreover, SAED is taken from >100 NPs which may all deviate in composition. Nevertheless, there is a clear deviation from pure Cu.

EDX mapping (Figure 4b) indicated a homogeneous distribution of Cu and Zn, again with excess Cu (92:8; Table T8). HAADF-STEM (Figure 4c) showed several nanoparticles, with the line scan across a small CuZn nanoparticle revealing a uniform distribution of Cu and Zn. The tiny Si signal likely originates from the polysiloxane glue used to seal the PLAL cell. In the future, we will thus use a stainless steel PLAL cell sealed by Kalrez O-rings.

**Figure 5:**
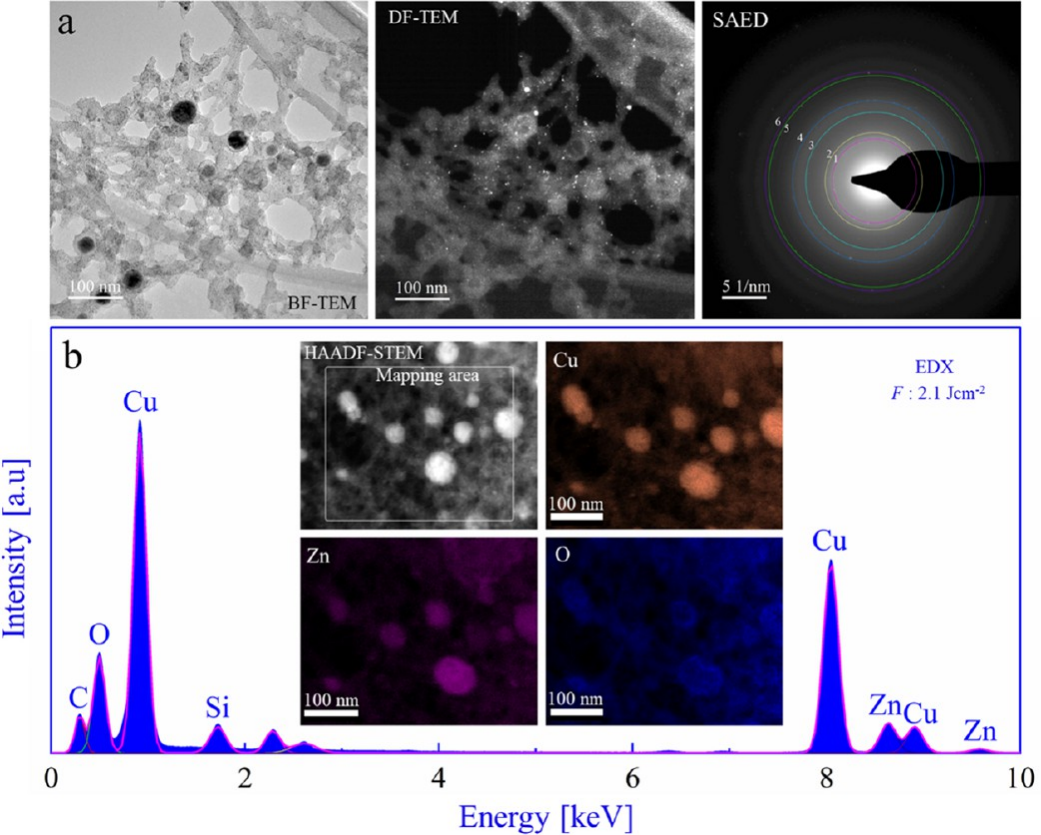
Transmission electron microscopy and EDX analysis of CuZn nanoparticles synthesized by fs-PLAL at *F*: 2.1 J cm^–2^. (a) BF-TEM, DF-TEM, and SAED (color circles are a guide for the eye); (b) EDX spectrum and HAADF-STEM with the elemental mapping area marked: Cu, Zn, and O maps are displayed.

BF-TEM micrographs of CuZn nanoalloys synthesized at 2.1 J cm^–2^ (Figure 5a) showed a bimodal size distribution similar to 1.6 J cm^–2^ (for a size distribution analysis see Figure S11cd). DF-TEM spectroscopy confirmed the crystallinity of the NPs. The SAED pattern was used to evaluate the lattice spacings (Table T9). The observed lattice distances again indicated Cu-rich/Zn alloys. Similar to the lower *F*, EDX mapping (Figure 5b) presented a homogeneous distribution of Cu and Zn with an average Cu/Zn ratio of 86:14 (Table T10).

**Figure 6:**
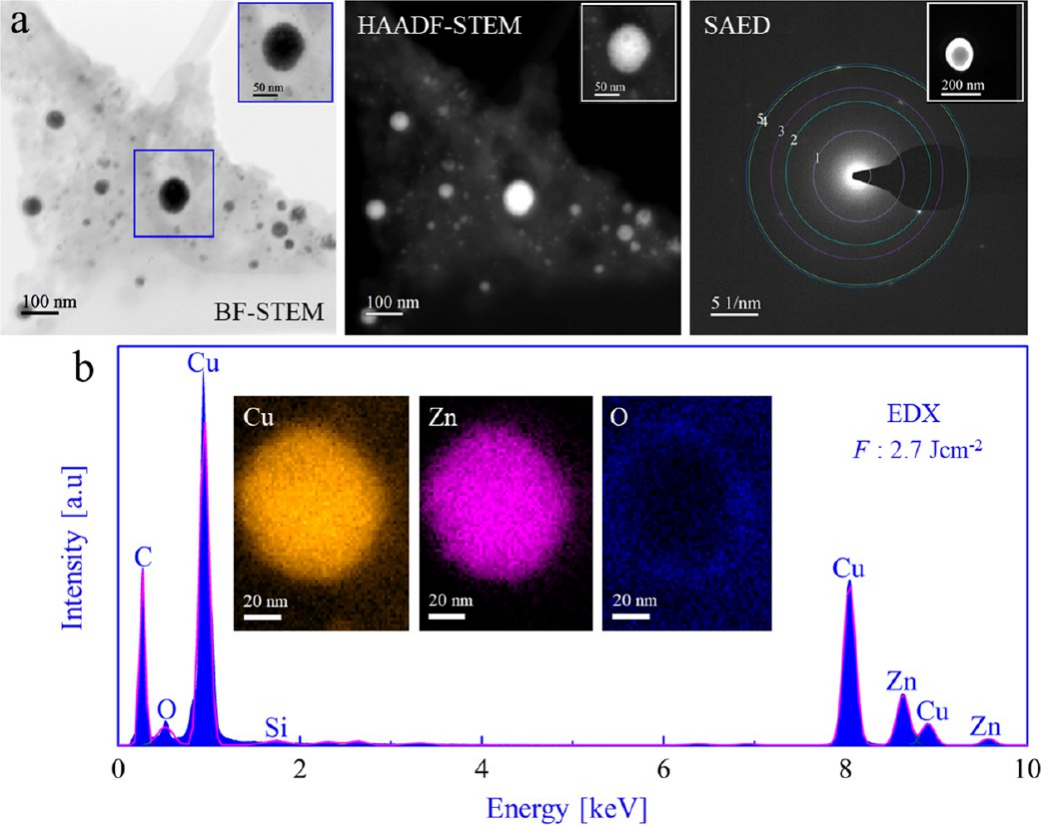
Transmission electron microscopy of CuZn nanoparticles synthesized by fs-PLAL at *F*: 2.7 J cm^–2^. (a) BF-TEM, HAADF-STEM, and SAED pattern; (b) EDX spectrum corresponding to a single CuZn NP with insets of EDX elemental mapping of Cu, Zn, and O.

Electron microscopy was also applied to CuZn NPs synthesized at 2.7 J cm^–2^ in order to evaluate the fluence effect on the size and elemental composition (Figure 6). BF-TEM again showed a bimodal size distribution, similar to that obtained at lower *F* (for a size distribution analysis see Figure S11ef). DF-TEM with bright contrast confirmed the crystallinity of both small and large NPs, and the SAED pattern (Figure 6) is in line with CuZn alloys. The observed lattice distances were similar to those of 1.6 J cm^–2^. The analysis of the diffraction spots is summarized in Table T11. EDX mapping of a single CuZn NP (Figure 6b) again showed a homogeneous elemental Cu/Zn distribution, but with higher Zn content (75:25; Table T12), nearly identical to the target composition. EDX analysis of several NPs produced at 2.7 J cm^–2^ revealed an average Cu/Zn ratio of 94:6. A diffraction pattern was additionally acquired from a larger collection of nanoparticles (BF-STEM, Figure S9a; SAED Figure S9b) to evaluate the average Cu/Zn ratio, also yielding compositions as mentioned above. Analysis of diffraction spots and corresponding lattice distances is summarized in Table T13. Further SAED analysis of another sample area (Figure S10b) indicated a Cu/Zn ratio as before; its analysis is summarized in Table T14. For better defect contrast, DF-TEM using bright spots located on the innermost ring were used (Figure S10b), so that crystalline nanoparticles showed a bright contrast (Figure S10cd).

**Figure 7:**
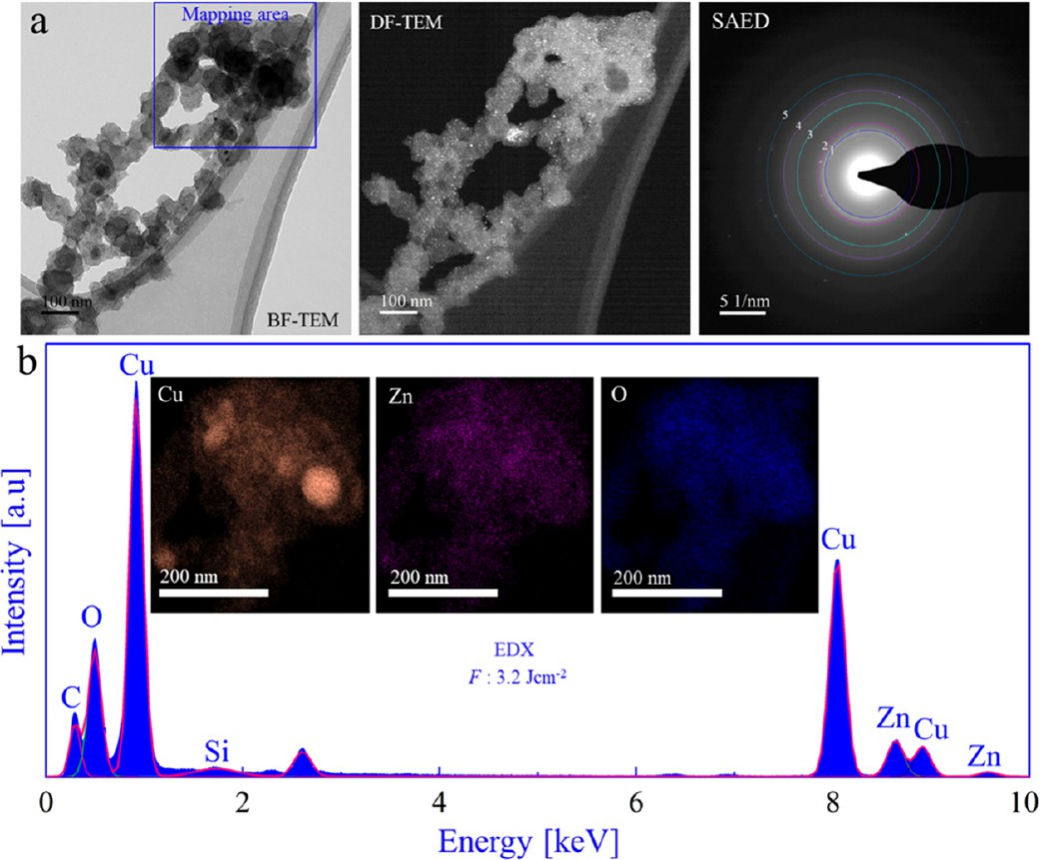
Transmission electron microscopy and EDX mapping of CuZn nanoparticles synthesized by fs-PLAL at *F*: 3.2 J cm^–2^. (a) BF-TEM, DF-TEM, and SAED pattern; (b) EDX spectrum and elemental mapping of Cu, Zn, and O.

BF-TEM micrographs of CuZn nanoalloys synthesized at 3.2 J cm^–2^ (Figure 7a) indicated a bimodal size distribution similar to 1.6, 2.1, and 2.7 J cm^–2^ (for size distribution analysis, see Figure S11gh). DF-TEM also showed a contrast change during electron beam shift, confirming CuZn crystallinity. SAED pattern analysis is summarized in Table T15 indicating Cu-rich/Zn alloys. Similar to the other applied laser fluences, EDX mapping (Figure 7b) displayed a homogeneous contribution of Cu and Zn with an average Cu/Zn ratio of 85:15 (Table T16).

**Figure 8:**
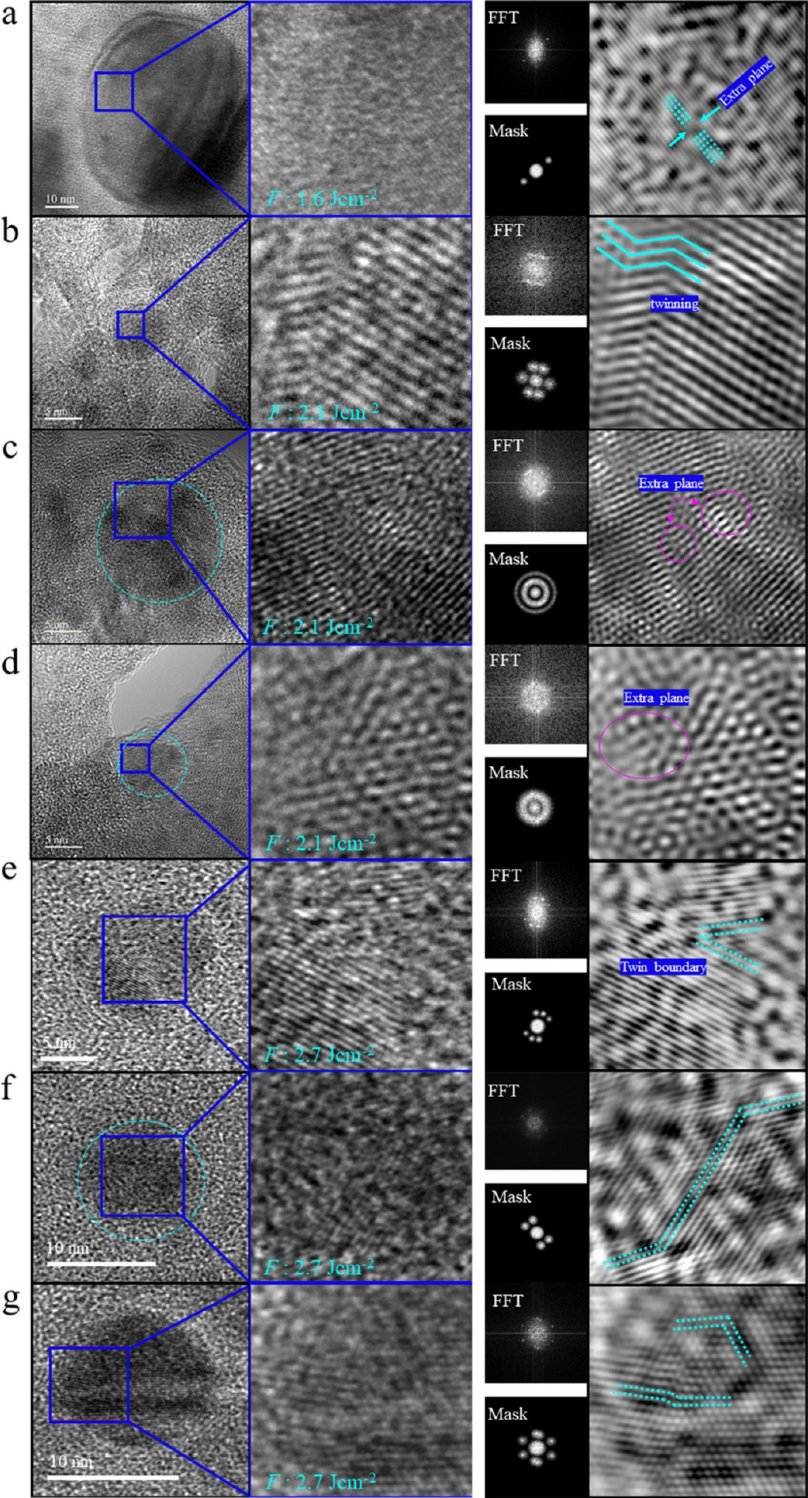
High-resolution transmission electron microscopy of CuZn nanoparticles synthesized by fs-PLAL: left: HRTEM and magnified view; right: FTT with spot or bandpass masks and IFFT. *F* is indicated in the images: (a) the inverse FFT shows an extra plane pointing to a stacking fault defect; (b) the IFFT shows twinning; (c) the IFFT shows SF defect; (d) the IFFT exhibits an extra plane corresponding to SF defect; (e–g) the inverse FFT displays twin boundaries.

To evaluate the crystal structure and defects of the CuZn NPs, HRTEM was applied (Figure 8). Interestingly, CuZn NPs produced at 1.6 J cm^–1^ showed a series of thickness fringes (Figure 8a), once more illustrating their 3D near-spherical shape. To improve visualization of the lattice structure, image processing was carried out via inverse fast Fourier transform (IFFT). Accordingly, an FFT of a specific area was calculated, and circular masks were placed on the bright spots, before an IFFT was calculated to reconstruct the image. In the cubic structure of CuZn NPs, irregularities in the atomic sequences can be discerned, pointing to a stacking fault (SF) defect (Figure 8a, IFFT).

Whereas HRTEM inspection of CuZn nanoparticles produced at 1.6 J cm^–2^ revealed few defects (Figure 8a), for those produced at 2.1, 2.7, and 3.2 J cm^–2^, many and different types of crystalline imperfections were observed. For example, HRTEM of a small CuZn NP (Figure 8b) showed nanotwinning (i.e., the twin lamellae spacing is <100 nm) and the crystal lattice on either side of the grain boundary exhibits mirror symmetry. Figure 8c presents stacking fault defects. Extra planes corresponding to an SF defect also appear in Figure 8d. Similarly, at higher *F*, the probability to find defective CuZn NPs was higher than that for 1.6 J cm^–2^. HRTEM of a small CuZn NP together with IFFT image processing (Figure 8e–g) also confirmed the existence of nanoscale twins.

**Figure 9:**
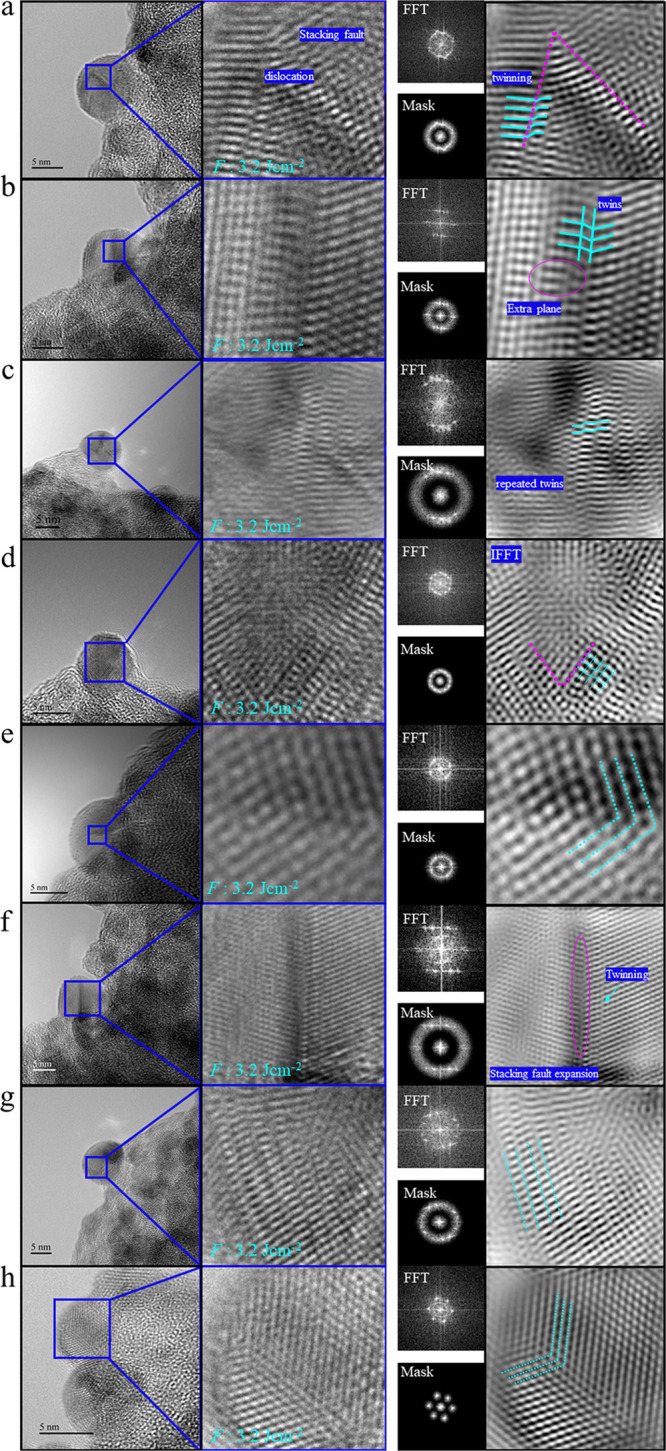
High-resolution transmission electron microscopy of CuZn nanoparticles synthesized by fs-PLAL at *F*: 3.2 J cm^–2^: left: HRTEM and magnified view; right: FTT with spot or bandpass masks and IFFT. The magnified areas show lattice fringes; the bandpass filter and spot masks were used for image processing and IFFT generation. IFFT confirms the existence and abundance of defects including stacking faults and twinning. (a) Stacking fault, dislocation, and twins; (b) combination of extra plane and twinning; (c) repeated twinning; (d) repeated twins and different grain boundaries; (e) twin boundary; (f) stacking fault expansion induce twinning at the grain boundary; (g) repeated twinning; (h) twin boundary.

At the highest *F* of 3.2 J cm^–2^ (Figure 9), due to higher thermal stress on crystalline NPs, the CuZn NPs were enriched in nanoscale twins, i.e., mostly repeated twins (Figure 9a–d,g). Similar twinned structures have been reported for materials under external mechanical forces, then called deformation twins.^61^ Deformation twins can result from the consecutive expansion of stacking fault planes (Figure 9f), thus the lower the intrinsic stacking fault energy (γ_ISF_) is, the easier it is to produce twins.^61^ Twin boundaries are also detected in Figure 9e,h. A complex combination of dislocation, stacking fault, and twins was evident in the magnified/IFFT (Figure 9a).

Accordingly, the abundance of twins in CuZn NPs at higher *F* values confirms a significant decrease in γ_ISF_. Furthermore, during the alloying process, different Cu/Zn ratios and atomic radii affect γ_ISF_. Since Cu and Zn have similar radii, lattice expansion is negligible; thus, different atomic contributions can be a further reason to affect the γ_ISF_. Correspondingly, the Cu stacking fault energy may decrease during alloying with different Zn atomic contributions.^62^ Presumably, isolated Cu and Zn NPs were formed in the plasma plum at the very early stages of CuZn target ablation; therefore, realloying of initial CuZn NPs may decrease γ_ISF_ and enhance twinning probability. Also, twinning in a cubic system such as Cu can easily occur when the surface energy is higher than the stacking fault energy; thus, twinning is a common deformation process of cubic structures to reduce surface energy.^63^

Previous studies of laser-derived ultrafine-grained Si NPs also showed twining defects,^49^ illustrating that short laser pulses generate defect-rich nanoparticles structures. This is due to the mechanism of PLAL, which comprises a series of phases, extending over many orders of magnitude in time (from fs to ms): ionization, plasma formation, bubble evolution, growth and collapse, ejection of nanomaterials into the liquid, and secondary processes.^39^ Most importantly, the fast femtosecond cooling rate^39^ creates many defects in nanostructures due to incomplete (partial) structural ordering. For femtosecond pulses, the temperature of the plasma at which nucleation begins is typically between 5000 and 7000 K.^64^ The plasma plum temperature upon femtosecond laser ablation of Cu in vacuum showed an increase up to 10 J cm^–2^ and after that a significant reduction of temperature was observed.^65^ The plasma excitation temperature for 1.6 J cm^–2^ was ∼9.100 K (without considering the liquid environment and the physicochemical properties of the target) and then increased up to ∼10.100 and ∼10.200 K for 2.1 and 2.7 J cm^–2^, respectively, and then decreased slightly to ∼9.900 J cm^–2^ for 3.2 J cm^–2^.^65^ These plasma temperature differences favor defect formation at higher fluences.

Concerning potential carbon deposits, laser interaction with organic precursors with high carbon numbers may trigger pyrolysis and photolysis reactions. However, PLAL in ethanol did not form carbon or graphite shells around the NPs, similar to previous HRTEM studies of fs-PLAL NiAu NPs^46^ and Si NPs.^49^

BF-TEM analysis of CuZn nanoalloy samples revealed a bimodal size distribution with the majority of particles between ∼2 and 8 nm and a smaller number of particles between ∼20 and 100 nm in size. Similar to DLS, smaller sizes were observed for *F*: 1.6 and 2.1 J cm^–2^ (mean 2.0 nm; first mode) than for *F*: 2.7 and 3.2 J cm^–2^ (mean 3 nm; first mode), whereas the second mode was larger for 1.6 and 2.1 J cm^–2^ (mean 37–38 nm vs 7–8 nm). As mentioned, likely due to agglomeration of NPs in the colloidal solution, the Stokes sizes determined by DLS were larger than the particle sizes observed by TEM. For completeness only, we determined the overall mean sizes from BF-TEM: 2.1, 2.4, 4.3, and 4.4 nm for 1.6, 2.1, 2.7, and 3.2 J cm^–2^, respectively. To evaluate whether the small or large NPs contribute more to catalytic performance, their contributions to the surface area (SA) and volume were calculated for the bimodal NPs. Figure S12 shows that for 1.6 J cm^–2^, smaller and larger (>50 nm) NPs contribute 50:50% to the total surface area of the catalyst, whereas it is 44:56, 70:30, and 60:40% for 2.1, 2.7, and 3.2 J cm^–2^ correspondingly. The number-weighted analysis of CuZn NPs produced at various fluences is summarized in Tables T17–T20.

### 3.3 XPS Analysis of HOPG Supported CuZn Nanoparticles and Catalytic Test Reaction

**Figure 10:**
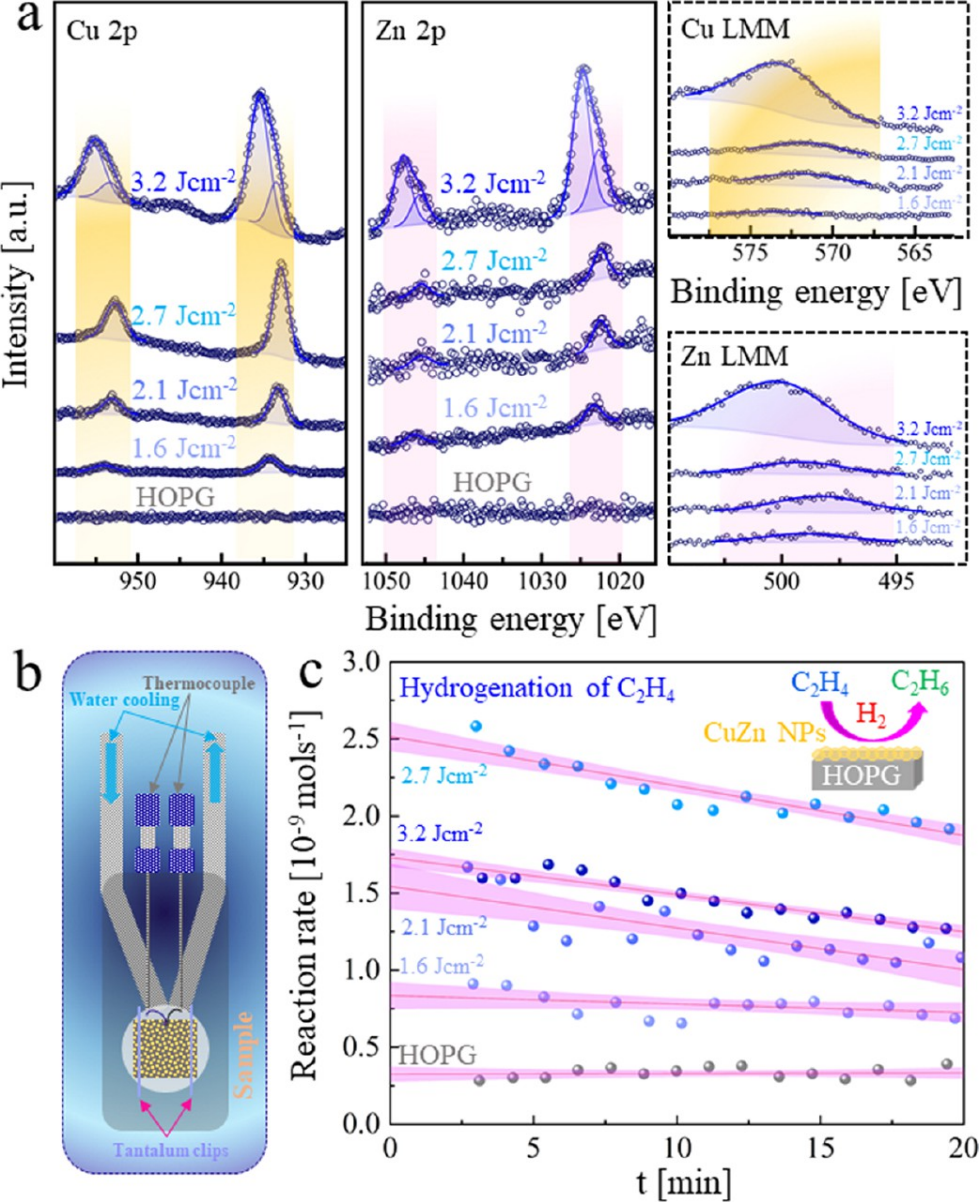
XPS analysis and catalytic activity of HOPG supported CuZn NPs. (a) XPS spectra of CuZn nanoparticles femtosecond laser-generated in ethanol (30 fs, 1 kHz, *N*: 1000) at fluences of 1.6, 2.1, 2.7, and 3.2 J cm^–2^, drop-casted on HOPG (spectra of pristine HOPG are included); blue solid lines are representative of the best fit; (b) schematic of the flow reactor cell used for ethylene hydrogenation on CuZn/HOPG nanoalloy catalysts; (c) reaction rate vs time of catalytic tests of CuZn nanoalloys and HOPG. The colored zone represents the 95% confidence interval of a linear fit.

After drop-casting the various colloidal CuZn NPs (*F*: 1.6, 2.1, 2.7, and 3.2 J cm^–2^) on HOPG, the chemical composition of the model catalyst was investigated by XPS, with spectra presented in Figure 10, including pure HOPG. For C 1s spectra (HOPG), see Figure S13.

Quantitative evaluation of the Cu 2p region (Figure 10a) indicated loadings of 0.1, 0.2, 0.6, and 1.5 at. % Cu for 1.6, 2.1, 2.7, and 3.2 J cm^–2^, respectively (which depend on the drop-casted amount). Compared to the CuZn target foil, the Cu 2p_3/2_ intensities of NP/HOPG are shifted to higher BEs around 933–934 eV, which is higher than the values expected for bulk Cu^0^ or Cu^+^. At the same time, the Cu 2p satellite peak typical for Cu^2+^ is only visible as a small feature in sample 3.2 J cm^–2^, while this species is absent in all other samples. The α′ values of 1847.2–1849.0 eV are closest to Cu_2_O, yet lower than the literature values of bulk Cu_2_O (explanation below). For 3.2 J cm^–2^, an additional high BE species at 935.5 eV, together with the appearance of the satellite feature, indicated Cu^2+^. Species at similar binding energy were also observed for laser ablation of pure Cu and assigned to CuO^66^ and Cu(OH)_2_.^67^ In agreement with SAED/EDX, the surface composition measured by XPS is copper rich with <0.1 at. % of zinc for 1.6, 2.1, and 2.7 J cm^–2^ and ∼0.4 at. % zinc for 3.2 J cm^–2^. Similarly, compared to the target foil, the Zn 2p_3/2_ peak of the NPs was also shifted to higher BEs of 1022.5–1023.0 eV, indicating oxidation. Accordingly, the Zn α′-values between 2009 and 2011.0 eV point to ZnO.^68^ While the BEs are higher than expected for bulk ZnO,^51^ they match well with ZnO NPs formed by PLAL. Again, an additional high energy species at 1024.9 eV is detected only for 3.2 J cm^–2^ and was assigned to Zn(OH)_2_.^68^

A shift of Cu BEs and α′ values (compared to bulk materials), as observed in this work, has been shown to occur with small particle sizes.^69^ Furthermore, considering the high surface sensitivity of XPS as well as the results of UV/vis, Raman, EDX, and SAED, the particles are likely to be metallic, but due to the air drying of samples on HOPG prior to XPS analysis, surface oxidation of CuZn NPs can occur.

To test the catalytic performance of femtosecond laser-generated CuZn NPs supported by HOPG, a simple test reaction, i.e., ethylene hydrogenation, was carried out. For this, the model catalysts were mounted in a small stainless steel reaction cell (reactor scheme shown in Figure 10b; volume of 4 mL), equipped with a thermocouple for temperature monitoring, external heating, and gas supply.^47,70^ Before the reaction, the catalysts were pretreated by oxidation (10 mL/min of 10% O_2_ in Ar, 1 atm, 150 °C, 60 min) to remove carbon contaminations and reduction (10 mL/min of 10% H_2_ in Ar, 1 atm, 150 °C, 60 min) to obtain a metallic state. The reaction was carried out at atmospheric pressure, at a constant temperature of 120 °C and using flow rates of 0.5, 7, and 2.5 mL/min of ethylene, hydrogen, and helium, respectively. The catalytic activity of pristine HOPG was measured as a reference. CuZn NPs/HOPG exhibited about 3–8 times higher activity than pure HOPG. The activity mostly follows the metal loading of the catalysts (1.6 < 2.1 < 2.7 J cm^–2^) except 3.2 J cm^–2^ which has the highest metal loading, yet is lower in activity than 2.7 J cm^–2^.

The lower activity of fs-PLAL CuZn NPs produced at 3.2 J cm^–2^ is likely related to a lower reducibility of Cu^2+^ and Zn(OH)_2_, observed by XPS for this sample only, or/and to more pronounced agglomeration, as evident from postreaction analysis (see next section). Still, the current initial activity measurements already demonstrate that PLAL-derived defective CuZn NPs can be employed as model catalysts.

### 3.4 Postreaction Analysis of Carbon Supported CuZn Nanoparticles

Finally, the stability of CuZn NPs under the reaction conditions was investigated. Accordingly, the CuZn NPs fs-synthesized at 1.6, 2.1, 2.7, and 3.2 J cm^–2^ were deposited on carbon-coated TEM-grids and subsequently exposed to the same reaction conditions (as in 3.3) in a thin quartz tube flow reactor (Figure S14), followed by postreaction TEM/HRTEM/SAED analysis (Figure S15). SAED confirmed that nanoparticles retained their crystallinity under the reaction conditions. Experimental lattice spacings are shown in T21–T24, corresponding to CuZn with a Cu contribution of ∼70–95% equivalent to that before reaction.

In the size range of 50 nm and above, growth and ripening were rarely observed. However, originally 2–3 nm sized NPs showed a tendency toward growth and coalescence. In general, depending on the reaction duration/temperature, deposition conditions, distance between NPs and their mobility, growth can be associated with either complete or incomplete nanoparticle diffusion. It is noteworthy that, in some cases, during the growth processes, small nanoparticles (NPs) exhibited a shape change from spherical to more complex morphologies, such as nanorods (Figure S15c). Since the reaction was conducted under atmospheric pressure, temperature might be an additional contributing factor to these shape modifications in NPs. Agglomeration/aggregation (sintering) was predominantly observed for 2–3 sized NPs, as depicted in Figure S15, and seems more significant for CuZn NPs synthesized at the highest fluence of 3.2 J cm^–2^. This can be attributed to the increased nanoparticle productivity, which in turn leads to larger deposition of nanoparticles on the HOPG substrate.

Such aggregation likely reduces the number of low-coordinated highly active (e.g., nanotwinned) sites, as aggregation apparently decreases the NP dispersion (surface to volume ratio), thereby reducing catalytic activity. Nevertheless, small isolated CuZn NPs were still observed, even in all samples prepared at various *F*, leading to both regions with isolated and aggregated/agglomerated NPs. Accordingly, under the current conditions, a fluence of 2.7 J cm^–2^ seems to be best for PLAL preparation of CuZn model nanocatalysts.

## Conclusions

CuZn alloy nanoparticles (NPs) were synthesized by femtosecond laser processing of Cu_0.70_Zn_0.30_ targets. EDX elemental mapping of CuZn nanoparticles showed a uniform Cu/Zn distribution, but the NPs were rich in Cu (∼70–95%), as further confirmed by XPS and SAED analysis. The analysis of ablated CuZn areas on the target revealed craters and self-organized surface structures.

HRTEM combined with IFFT image processing visualized the crystal structures and defects of the CuZn nanoalloy particles. For lower *F* of 1.6 J cm^–2^, additional atomic planes were observed; while at higher *F* of 2.1, 2.7, and 3.2 J cm^–2^, nanoparticles were enriched in dislocations and multiple twins with visible grain boundaries. This is in line with higher collision rates of primary nanoparticles at higher *F* and incomplete ordering due to fast cooling/solidification characteristics of ultrashort pulses, creating various crystalline imperfections. Twinning was dominant when the laser fluence increased from 2.1 up to 3.2 J cm^–2^. The observed increase in twinning can be attributed to several factors: first, ultrashort pulses and higher laser fluence facilitate twin formation by rapidly cooling the primary nanoparticles in the high-pressure/high-temperature plasma, thereby reducing the stacking fault energy. Second, the realloying of isolated Cu and Zn nanoparticles, generated in the plasma plum, can also reduce the nanoparticle stacking fault energy. Third, surface energy minimization of cubic metal nanoparticles resulted in a systematic formation of repeated self-organized nanotwinned structures, creating low-coordinated catalytically active sites.

The nanoparticle sizes also increased upon increasing *F*, as observed by dynamic light scattering analysis of the NP colloids and by TEM. BF-TEM imaging indicated bimodal distributions, typically around 2–3 nm and 20–40 nm. Calculating the surface area indicated that the smaller NPs (<50 nm) still dominated the overall metal surface area of the model catalysts: 60 to 70% at higher *F* (2.7 and 3.2 J cm^–2^) and 40 to 50% at lower *F* (1.6 and 2.1 J cm^–2^). The abundance of the first mode of nanoparticle size (2–3 nm) was higher at the highest *F*.

CuZn nanoparticles drop-cast on HOPG supports were used for ethylene hydrogenation to ethane, employing a flow microreactor at atmospheric pressure, and similar technological catalysis. The activity mostly followed metal loading of the catalysts. However, 3.2 J cm^–2^ with the highest metal loading and most defects did not yield the highest activity. This can be rationalized by a higher amount of oxidation and stronger agglomeration under the reaction conditions. Postreaction TEM analysis indicated aggregation and agglomeration of small NPs (2–3 nm in size), while larger NPs remained unchanged. Although aggregation occurred for all samples prepared by different laser fluences, it was more pronounced for the 3.2 J cm^–2^ sample, since a high number of small NPs was initially present. Thus, the increase in size (decrease in dispersion) and a decrease in the number of active sites may explain the smaller activity. Nevertheless, the observed activity confirmed that defect-rich CuZn nanoparticle model catalysts, prepared by fs-laser ablation, are indeed worthwhile for further studies. Clearly, the challenge is to optimize the PLAL to obtain more uniform nanoparticle sizes and compositions.

## Supporting Information Available

Laser fluence evaluation, characterization techniques, analysis of the CuZn target and of (supported) CuZn nanoalloys, and postreaction analysis (PDF)
