# Supplementary material for: Defect-Rich CuZn Nanoparticles for Model Catalysis Produced by Femtosecond Laser Ablation
Source: ACS Appl Mater Interfaces. 2024 Jun 27;16(29):38163–76. doi: 10.1021/acsami.4c07766 (PMC11284744; doi:10.1021/acsami.4c07766)
Supplement: Supplementary file 1 — am4c07766_si_001.pdf [file am4c07766_si_001.pdf]

# Supporting Information

# Defect-rich CuZn Nanoparticles for Model Catalysis Produced by Femtosecond Laser Ablation

Niusha Lasemi [1], Thomas Wicht [1], Johannes Bernardi [2], Gerhard Liedl [3], Günther Rupprechter [1*]

[1] Institute of Materials Chemistry, Technische Universität Wien, 1060 Wien, Austria.

[2] University Service Center for Transmission Electron Microscopy, Technische Universität Wien, 1020 Wien, Austria

[3] Institute of Production Engineering and Photonic Technologies, Technische Universität Wien, 1060 Wien, Austria.

**Correspondence to:** guenther.rupprechter@tuwien.ac.at

**Note:**

The PDF file contains supplementary discussion including figures and tables related to CuZn target analysis (after ablation) and (supported) CuZn nanoalloys. Corresponding references related to the supplementary information are located at the end of the PDF.

## 1. Laser fluence evaluation

The squared diameter of the ablated area ($D^2$) on CuZn at various $F$ was evaluated by optical microscopy (Zeiss AxioVision software). For this evaluation, an average of 30 craters was considered. The laser fluence ($F$) was varied to find the optimized $F$ for the production of defect-rich nanoparticles and the highest ablation rate. Assuming a Gaussian beam, equation 1[1] can be used to correlate $D^2$ with the Gaussian beam radius ($w_0$), pulse energy ($E$) and threshold energy ($E_{th}$).

Eq. 1 $$D^2 = 2w_0 \ln(E/E_{th})$$

Squared diameter of the ablation area versus laser energies is shown in Figure S1. $w_0$ was evaluated to be 77.5 µm and used to calculate $F$ by equation 2. A threshold energy of 0.06 mJ was measured for CuZn ablation with 1000 pulses in ethanol. The measured $F$ and crater diameters are summarized in Table T1. To measure $D^2$, the area of an oval was used considering short and long axes, since the craters produced on the CuZn surface in ethanol were not round.

Eq. 2 $$F = 2E/\pi w_0^2$$

**Table T1.** Calculated $F$ and measured crater diameters for CuZn target femtosecond laser-ablated in ethanol.

| $P$ [mW] | $F$ [Jcm$^{-2}$] | crater diameter [µm] |
|---|---|---|
| 100 | 1.0 | 75.5 |
| 150 | 1.6 | 85 |
| 200 | 2.1 | 106 |
| 250 | 2.7 | 112.5 |
| 300 | 3.2 | 127 |

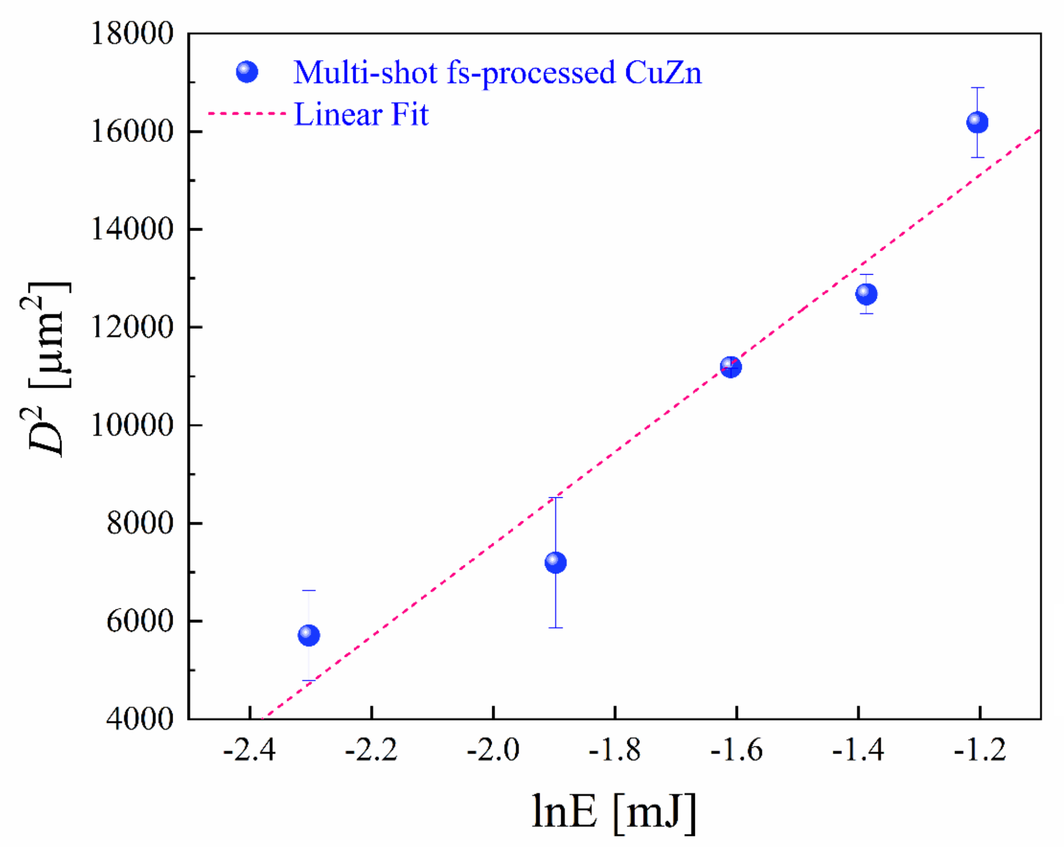


**Figure S1**. Squared diameter of the ablation area versus laser energies (ethanol, 30 fs, 1 kHz, $N = 1000$), based on optical microscopy.

## 2. Characterization techniques

### 2.1 Scanning electron microscopy

A Schottky FEG-SEM (Field emission gun-scanning electron microscope) from TESCAN MIRA3 was used for the imaging of craters with a secondary electron (SE) detector operated at 20 kV, with a spot size of 35 nm and a working distance of 14.95 mm. A MIRA3 chamber with a 5-axis fully motorized stage was used for energy dispersive X-ray spectroscopy (EDX) analysis. A silicon drift detector (SDD) with a thin film Motextek AP1.3 window type was applied at a probing current ($i_p$) of 10 nA for elemental mapping and chemical identification of CuZn targets by using MaxView and IDfix software, respectively. SEM image processing was carried out by ImageJ software to extract FFT (fast Fourier transform) and periodicity profile for defining laser-induced periodic surface structures (LIPSS).

### 2.2 Profilometry

A DektakXT stylus profilometer (Bruker) device with a conically shaped diamond tip (radius of 2 μm) was used to measure the ablation depth and to generate three-dimensional (3D) maps of the CuZn craters. For analysis, a stylus force of 3 mg was used. To enhance the quality of 3D mapping, profilometry with a resolution of 12 μm per trace was applied.

### 2.3 Micro-grazing incidence X-ray diffraction (μ-GIXRD)

μ-GIXRD analysis (Empyrean, PANALYTICAL) was done on pristine and laser-treated areas of CuZn to analyze crystallinity, using Cu-Kα radiation (1.54 Å), operated at 45 kV and 40 mA. A motorized XYZ stage and a charge-coupled device (CCD) camera allowed to move the target, so to expose a selected area to the focused X-ray beam. For higher surface sensitivity, the incidence angle (ω) was fixed at 6°. For micro-analysis, the system was equipped with a 2D-detector position (GaliPIX3D, Malvern PANalytical), a parallel beam x-ray collimator and a micro-nozzle (diameter of 300 μm). Data analysis was performed by using HighScore software and its corresponding International Center for Diffraction Data (ICDD) database.

## 2.4 Dynamic light scattering (DLS)

DLS was operated by means of a compact goniometer system (ALV/CGS-3) which includes a helium–neon (He-Ne) laser that operates at 632.8 nm with a power of 22 mW. DLS measurements of colloidal CuZn nanoparticles were done at room temperature and angle of (θ : 90°) for a better signal-to-noise ratio. To measure polydispersity (PDI), Cumulant analysis (simple fit approach) was applied. To measure the mean Stokes radius ($R_s$), translational diffusion ($D_{trasn}$), rotational diffusion ($D_{rot}$) translational friction ($f_{trans}$) and rotational friction ($f_{rot}$); Contin analysis was applied where the intensity autocorrelation function curve was fitted by regularized fit.

## 2.5 Confocal micro-Raman spectroscopy

A Horiba XploRA™ INV equipped with a thermoelectrically cooled charge-coupled device (CCD) detector and a fully XY motorized stage was used for micro-Raman analysis at room temperature. An excitation source (diode laser) at 532 nm and 25% of the maximum power ($P_{max}$ : 100 mW) was used for Raman scattering. An optical microscope (Nikon Eclipse TiU) was connected to the Raman system to focus the beam on a selected zone. After full calibration of the system, a 40x objective was applied. Spectra of CuZn targets were recorded between 0 and 3000 $cm^{-1}$ by applying a holographic grating of 1200 grooves/mm, a slit of 200 μm and a hole of 100 μm. To improve accuracy, five areas of pristine target and craters were analyzed. To increase the signal-to-noise ratio, an acquisition time of 1 second and an accumulation of 30 spectra were chosen, with spectra collection and background subtraction performed by HORIBA Scientific's LapSpec 6 spectroscopy suite software. The micro-Raman spectroscopy of colloidal CuZn NPs was done using μ-dish holders (Ibidi) with a glass bottom and a diameter of 35 mm.

## 2.6 UV/Vis spectroscopy

To study the optical properties of colloidal CuZn nanoparticles, the colloidal solutions were injected into a high-precision quartz cell (10 mm path length) purchased from Hellma Analytics and analyzed using a PERKIN-ELMER Lambda20 UV/Vis spectrophotometer with two radiation sources including a deuterium lamp and a halogen lamp, covering the working wavelength range of the spectrometer (190-1100 nm, ±0.3 nm). The spectrophotometer consists of a holographic concave grating monochromator with 1053 lines/mm. The collection of spectra including blank and CuZn NPs was performed at scan speeds of 240 nm/mm in the wavelength range of 190-800 nm. UV Winlab software was used to collect and analyze the spectra.

### 2.7 Transmission electron microscopy

CuZn nanoparticle analysis was carried out by using transmission electron microscopy (TEM), on a FEI Tecnai F20 S-TWIN. High-resolution transmission electron microscope (HRTEM) was operated at 200 kV. Dark field (DF) and bright field (BF) TEM were applied to investigate the size and shape of CuZn nanoalloys. A high-angle annular dark-field (HAADF) detector and scanning transmission electron microscopy (STEM) were applied for EDX mapping with a take-off angle of 15°. FFT (fast Fourier transform), IFFT ( Inverse Fourier transform), and selected area electron diffraction (SAED) patterns were studied by the Gatan microscopy suite software (DigitalMicrograph). Lattice distances (d) and Miller indices (*hkl*) were found by using HighScore software (ICDD database).

### 2.8 X-ray photoelectron spectroscopy

X-ray photoelectron spectroscopy (XPS) was performed in an ultra-high vacuum (UHV) system with a base pressure of $\leq 10^{-8}$ mbar including a high-intensity non-monochromatic Al/Mg dual anode X-ray source (Specs XR50 GmbH) and a hemispherical energy analyzer (Phoibos 100©) with multichannel plate detector.[2] All spectra were taken at room temperature, using the Al anode with Kα radiation at 1486.6 eV, an emission angle of 0° and using an analyzer pass energy of 20 eV.

The pristine CuZn foil was first cleaned by sputtering (1.5 keV, $5\times10^{-6}$ mbar $Ar^+$ at room temperature) to remove the oxide layer and carbon contamination. The colloidal CuZn nanoparticles in ethanol were sonicated and deposited by drop casting method on highly oriented pyrolytic graphite (HOPG). The NP/HOPG samples were analyzed as-is. Further, a clean (freshly cleaved) HOPG crystal was used as a reference.

The pristine and ablated CuZn foil was calibrated to the Fermi edge at 0.0 eV. For pure HOPG and NPs/HOPG, the binding energy in the spectra was referenced to the C 1s peak at 284.3 eV.[3]

## 3. CuZn target analysis

CuZn targets were ablated in ethanol by a near-infrared femtosecond laser at various fluences. For this, polished CuZn samples were placed at the bottom of a glass cell filled with ethanol and 1000 shots were applied. All experiments were performed at a focal point without exerting defocusing. Several techniques were applied to study CuZn targets before and after femtosecond laser processing and the nanoparticles. To visualize the three-dimensional (3D) shapes of the craters and to measure their diameters, profilometry and optical microscopy were applied.

To observe crater morphology and elemental composition/distribution, SEM/EDX-mapping was used. Moreover, fast Fourier transform (FFT) patterns were applied to analyse nanoripple deviations. The chemical composition of CuZn targets was also studied by confocal micro-Raman spectroscopy. The surface/crater crystallinity was examined by μ-GIXRD.

### 3.1 Profilometry

CuZn craters were studied by profilometry and optical microscopy to measure the depth and to calculate the ablation volume and specific ablation rate (Figure S2a-e). Due to the conical shape of the ablation craters, the volume of a cone was considered. By increasing *F*, the crater depth was decreased, while the crater diameter increased. Ablation at 1 $Jcm^{-2}$ led to the highest specific ablation rate, while the highest ablation volume was observed at 3.2 $Jcm^{-2}$.

The physiochemical properties of the liquid and target can both affect the energy deposition on the surface. Thus, knowing the optimum *F* for nanoparticle synthesis in liquids is of importance. In previous studies, Au showed a maximum ablation efficiency for *E*: 200 μJ.[4] Also, for femtosecond processing of $SiO_2$[5] and Au-coated Ni[6], 250 μJ was selected for the highest ablation rates.

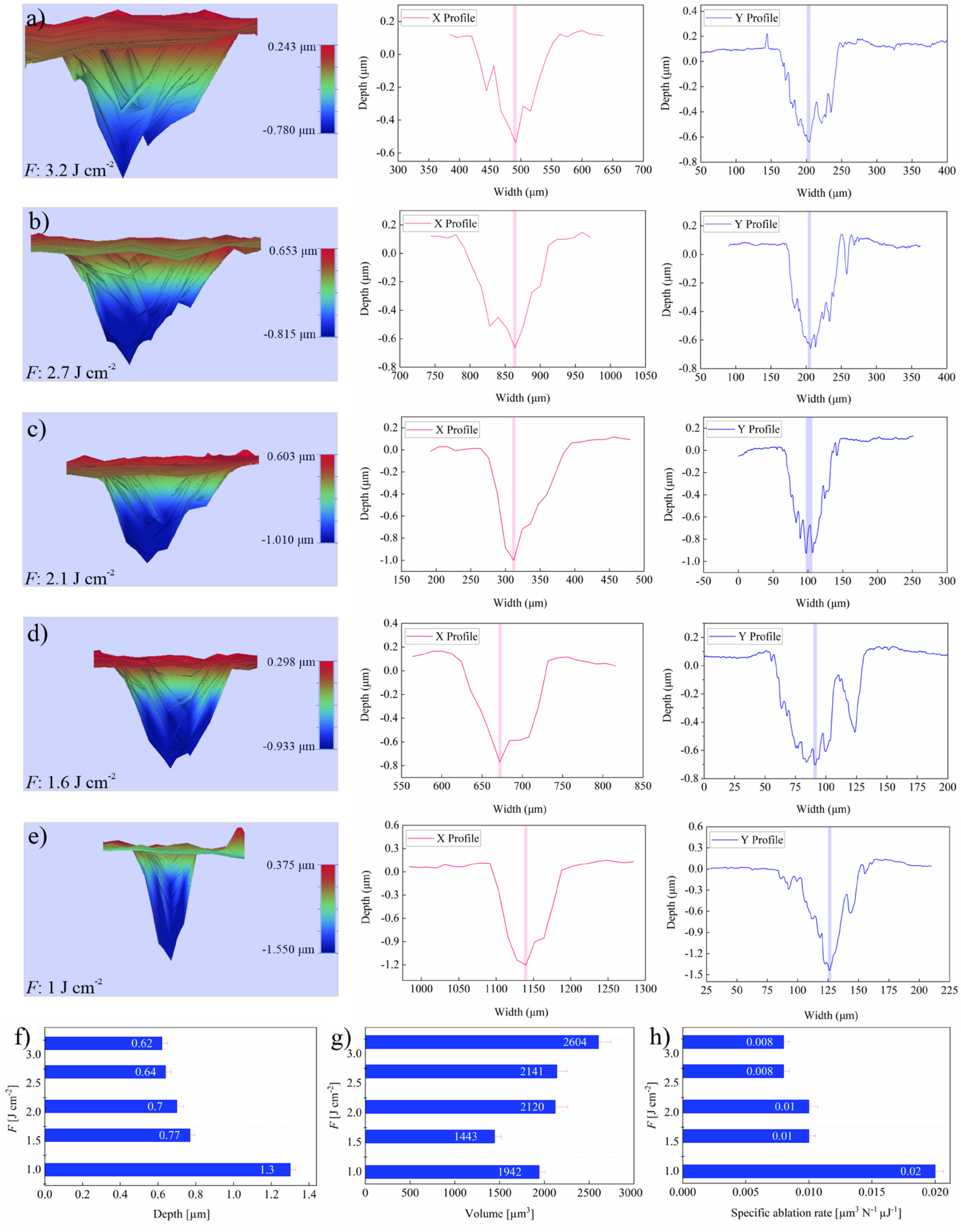


**Figure S2.** Three-dimensional mapping of craters on CuZn processed by femtosecond laser radiation at various laser fluences (30 fs, 1 kHz) in ethanol with their corresponding XY profiles; (a) *F*: 3.2 Jcm$^{-2}$; (b) *F*: 2.7 Jcm$^{-2}$; (c) *F*: 2.1 Jcm$^{-2}$; (d) *F*: 1.6 Jcm$^{-2}$; (e) *F*: 1.0 Jcm$^{-2}$; (f) Crater depth *versus F*; (g) Crater volume *versus F*; (h) Specific ablation rate *versus F*.

Accordingly, nonlinear processes (such as filamentation) could not negatively affect nanoparticle productivity, since higher ablation volume was observed with increasing of $F$ up to 3.2 $Jcm^{-2}$. 3D imaging showed multiple scattering at the bottom of the craters, which is more pronounced and chaotic at higher laser fluences, creating multiple internal reflections, liquid turbulence and beam deviation due to interaction with ejected particles.[7]

### 3.2 SEM/EDX and FFT

The FEG-SEM images (Figure S3) show pristine CuZn surface morphologies and craters. The alloying elements in the bulk were confirmed by EDX, as shown in Figure S3. The table of EDX results (Table T2) shows minor oxidation (native oxide) and carbonization (adventitious) of the CuZn surface before and after applying laser fluences. The distribution of elements (Cu, Zn, and O) is illustrated by EDX mapping using colour coding. All elements were uniformly distributed over the CuZn surface and craters.

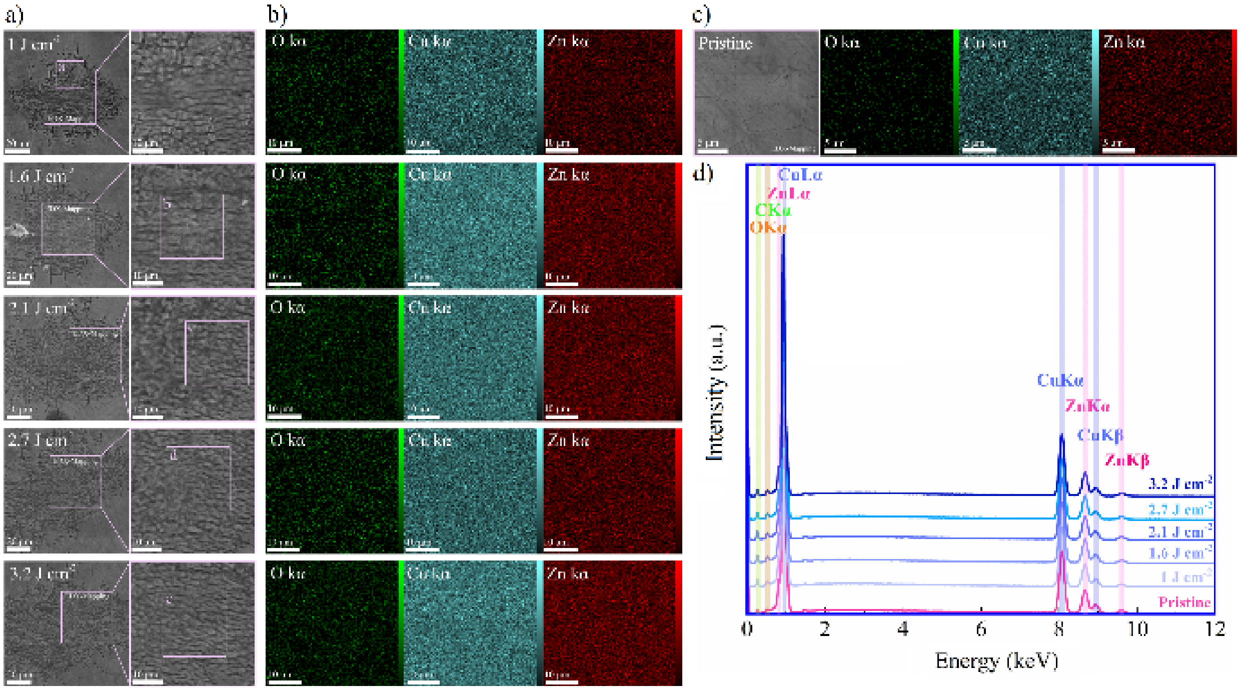


**Figure S3.** (a) FEG-SEM images; (b) EDX-elemental mapping; (c) pristine target and (d) EDX spectra of the craters created by CuZn femtosecond-processing at various $F$ in ethanol.

**Table T2.** EDX atomic percent (at.%) analysis of the CuZn target: pristine vs femtosecond-processed at various *F* [$Jcm^{-2}$] in ethanol.

| | Cu at.% | Zn at.% | C at.% | O at.% | Ratio Cu/Zn |
|---|---|---|---|---|---|
| Pristine | 45.60 | 17.00 | 26.30 | 11.10 | 2.68 |
| *F*: 1.00 | 32.20 | 12.60 | 41.20 | 14.00 | 2.56 |
| *F*: 1.60 | 33.20 | 12.70 | 37.20 | 16.90 | 2.61 |
| *F*: 2.10 | 33.60 | 12.10 | 41.20 | 13.10 | 2.78 |
| *F*: 2.70 | 35.20 | 13.40 | 38.20 | 13.20 | 2.63 |
| *F*: 3.20 | 34.80 | 12.93 | 37.20 | 15.07 | 2.69 |

FEG-SEM images of CuZn craters (Figure S4) revealed the morphology of laser induced periodic surface structures (LIPSS), with a periodicity of low spatial frequency LIPSS (LSFL) near the laser wavelength ($\lambda/2 \leq \Lambda_{LSFL} \leq \lambda$). The LIPSS orientations on CuZn were perpendicular to the horizontal beam polarization, similar to femtosecond processed Au-coated Ni in various liquids.[8]

FFT images showed that an increase in laser fluence affects the deviation angle of nanoripples. The lowest and highest deviation angles of 5.3° and 8.6° were measured for 1 and 3.2 $Jcm^{-2}$, correspondingly. In general, laser ablation in liquids causes liquid turbulence[9] at the liquid/solid interface which can be a reason for angular deviation.[8] Thus, the increased *F* may also increase the turbulences since more energy can be delivered to the liquid by high-intensity femtosecond pulses, which may initiate Marangoni bursting[10] due to liquid evaporation.

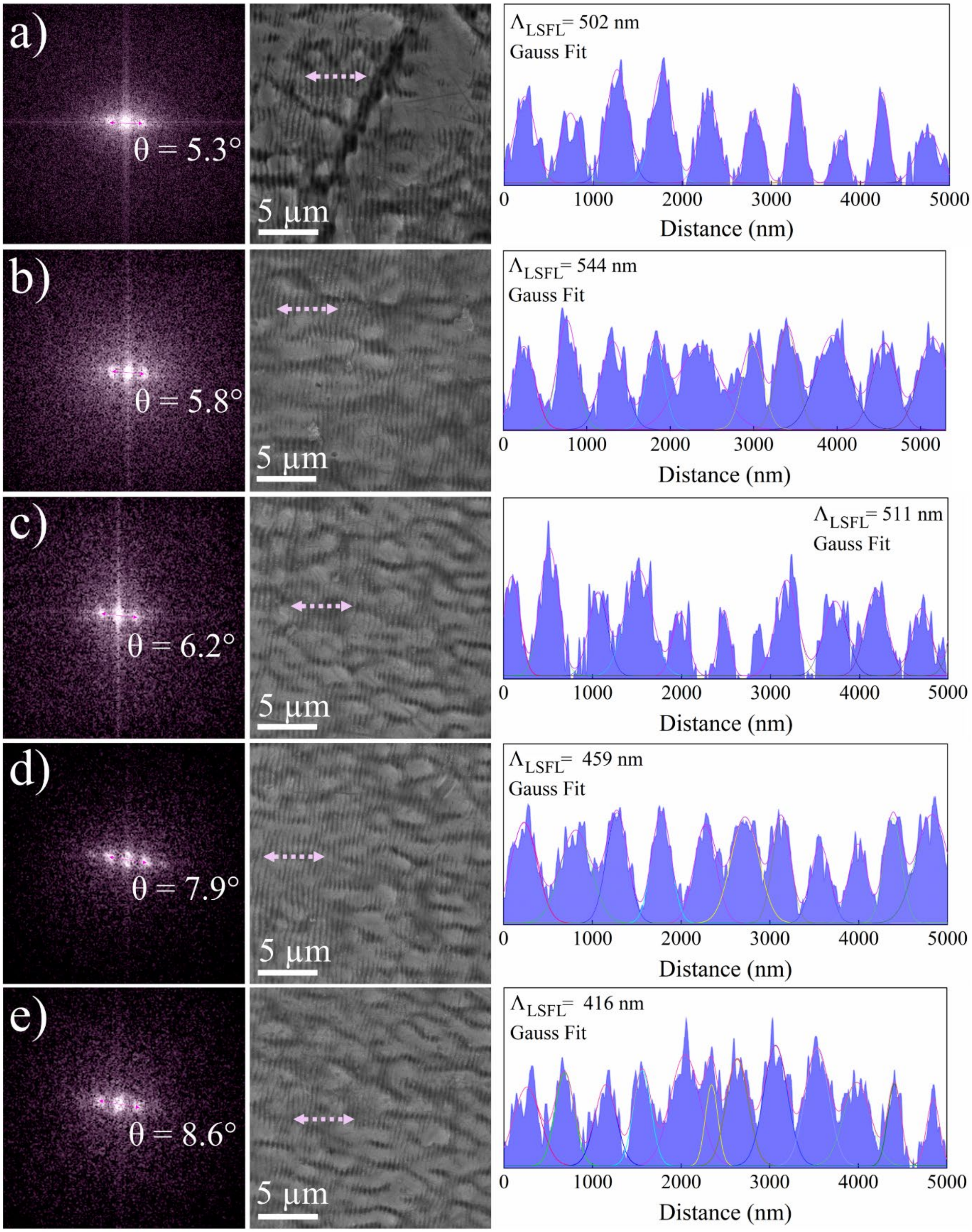


**Figure S4.** Magnified SEM images of craters (middle), FFT (left) and corresponding LIPSS profiles (right) of CuZn processed by femtosecond laser radiation in ethanol at various laser fluences. (a) *F*: 1.0 $Jcm^{-2}$; (b) *F*: 1.6 $Jcm^{-2}$; (c) *F*: 2.1 $Jcm^{-2}$ (d); *F*: 2.7 $Jcm^{-2}$; (e) *F*: 3.2 $Jcm^{-2}$.

### 3.3 Micro-Grazing Incidence X-ray Diffraction

Williamson–Hall plots[11] were applied to evaluate the microstrain (ε) and crystallite size ($l_{WH}$) for areas laser-processed on CuZn at various $F$ (Figure S5, Table T3). Tensile forces occurred since microstrain values exhibited positive slopes. The highest microstrain and crystallite size were observed for 2.7 Jcm$^{-2}$, however, the increase of ε and $l_{WH}$ with respect to $F$ was not linear.

Diffraction patterns of CuZn targets were acquired by micro-GIXRD, both for non-irradiated and irradiated zones for various $F$ (Figure S5a). CuZn peaks corresponded to JCPDS card no. 04-006-2621. Moreover, pristine areas showed intense diffraction peaks related to cubic $Cu_{0.7}Zn_{0.3}$ alloy. Figure S6 shows that the CuZn (111), (200) and (222) peaks of areas treated at $F$ : 1 Jcm$^{-2}$ compared to other $F$ were shifted to smaller angles. Since the applied $F$ was slightly higher than the threshold fluence ($F_{th}$ : 0.65 Jcm$^{-2}$) of CuZn in ethanol, lattice expansion could be a possible reason for the shift.

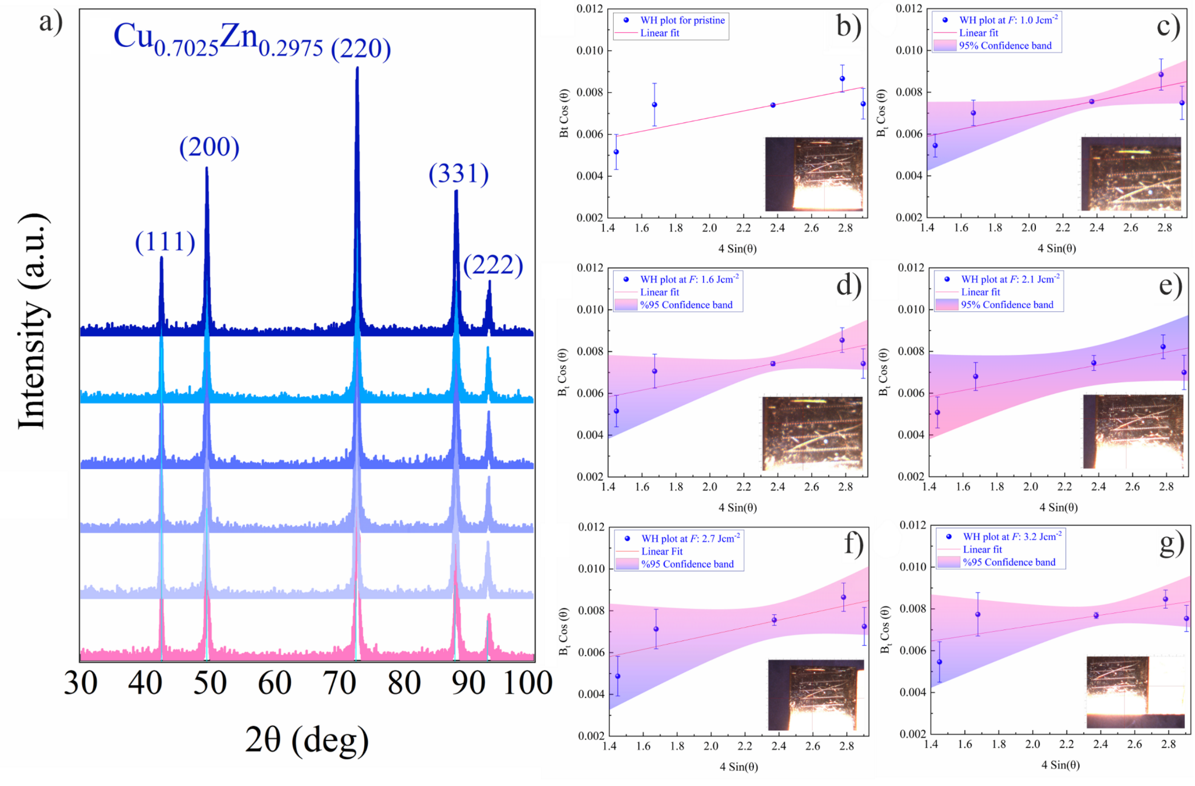


**Figure S5.** (a) μ-GIXRD results, both for pristine and femtosecond laser-ablated CuZn for various $F$ in ethanol. Williamson-Hall plots: (b) Pristine; (c) $F$: 1.0 Jcm$^{-2}$; (d) $F$: 1.6 Jcm$^{-2}$; (e) $F$: 2.1 Jcm$^{-2}$; (f) $F$: 2.7 Jcm$^{-2}$; (g) $F$: 3.2 Jcm$^{-2}$. The colored zone represents the 95% confidence interval for a linear fit.

**Table T3.** μ-GIXRD results for CuZn processed by femtosecond laser in various *F*.

| *F* [$Jcm^{-2}$] | ε | $l_{WH}$ [nm] |
|---|---|---|
| - | $1.46 \times 10^{-3}$ | 35 |
| 1.0 | $1.55 \times 10^{-3}$ | 36 |
| 1.6 | $1.53 \times 10^{-3}$ | 37 |
| 2.1 | $1.38 \times 10^{-3}$ | 36 |
| 2.7 | $1.62 \times 10^{-3}$ | 40 |
| 3.2 | $1.21 \times 10^{-3}$ | 30 |

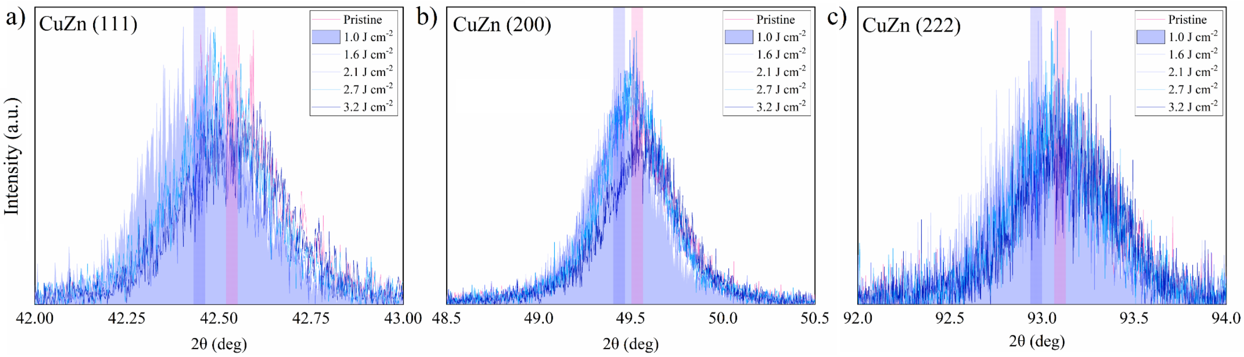


**Figure S6.** Magnified regions of μGIXRD of CuZn, for pristine and femtosecond laser-ablated areas in various *F*, presenting (a) CuZn (111); (b) (200), and (c) (222).

Micro-Raman studies of CuZn, both for pristine and for areas treated at various *F*, were recorded between 0 to 3000 $cm^{-1}$ at a laser excitation wavelength of 532 nm, as presented in Figure S7 and Table T4. Cu oxidation increased upon increasing the laser fluences up to 2.1 $Jcm^{-2}$ and after that decreased to the oxidation level similar to 1 $Jcm^{-2}$. At 2.1 $Jcm^{-2}$, $Cu_2O$ exhibited a distinguished doublet with respect to other areas. A weak signal at ~400 $cm^{-1}$ (Figure S7) also corresponds to the four-phonon mode (3Eu + T1u) related to $Cu_2O$ formation.[12]

Moreover, a very low intensity and broad peak of graphitization (D/defect and G/graphite bands) was observed in all areas without the existence of a 2D-band (two phonon lattice vibration) ~2500-2700 $cm^{-1}$, indicating a minor amount of surface carbonization.

### 3.4 Confocal micro-Raman spectroscopy

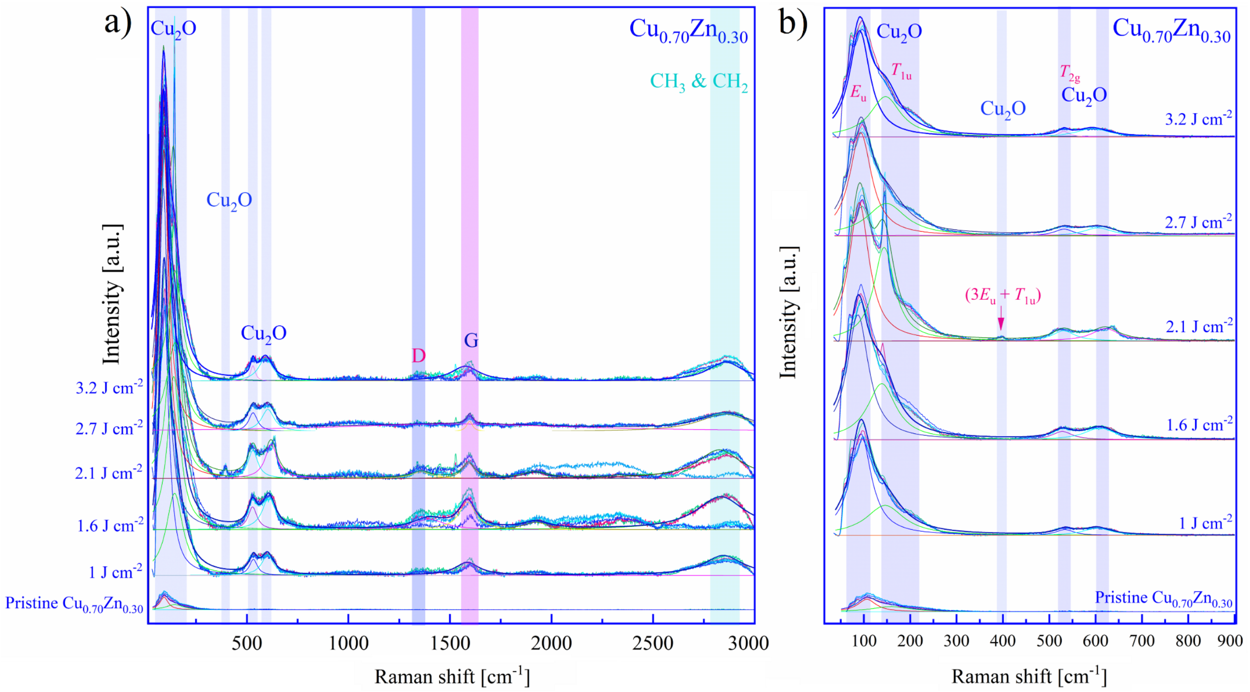


**Figure S7**. (a) Confocal micro-Raman spectroscopy of CuZn, pristine and femtosecond laser-ablated areas at various *F*; (b) Magnified region related to $Cu_2O$ peaks. A Lorentz fit was applied to all spectra. Cumulative fits and corresponding de-convoluted peaks are applied based on the best $R^2$. Pristine CuZn ($R^2$: 0.95); *F*: 1 Jcm$^{-2}$ ($R^2$: 0.97); *F*: 1.6 Jcm$^{-2}$ ($R^2$: 0.97); *F*: 2.1 Jcm$^{-2}$ ($R^2$: 0.96); *F*: 2.7 Jcm$^{-2}$ ($R^2$: 0.97) and *F*: 3.2 Jcm$^{-2}$ ($R^2$: 0.96). The magnified region corresponds to the signature peaks of $Cu_2O$.

**Table T4**. Evaluated Raman intensities of pristine CuZn (*F* : 0 Jcm$^{-2}$) and areas femtosecond ablated in ethanol at various *F*.

| *F* [Jcm$^{-2}$] | $Cu_2O$ 89-93 cm$^{-1}$ | $Cu_2O$ 141-148 cm$^{-1}$ | $Cu_2O$ 529-621 cm$^{-1}$ |
|---|---|---|---|
| 0.0 | 284 | 119 | - |
| 1.0 | 6301 | 1857 | 264-455 |
| 1.6 | 7739 | 3474 | 517-730 |
| 2.1 | 8585 | 5800 | 635-774 |
| 2.7 | 6406 | 2021 | 428-504 |
| 3.2 | 6583 | 2496 | 354-529 |

## 4. CuZn nanoparticle analysis

### 4.1 Micro-Raman spectroscopy of colloidal CuZn NPs

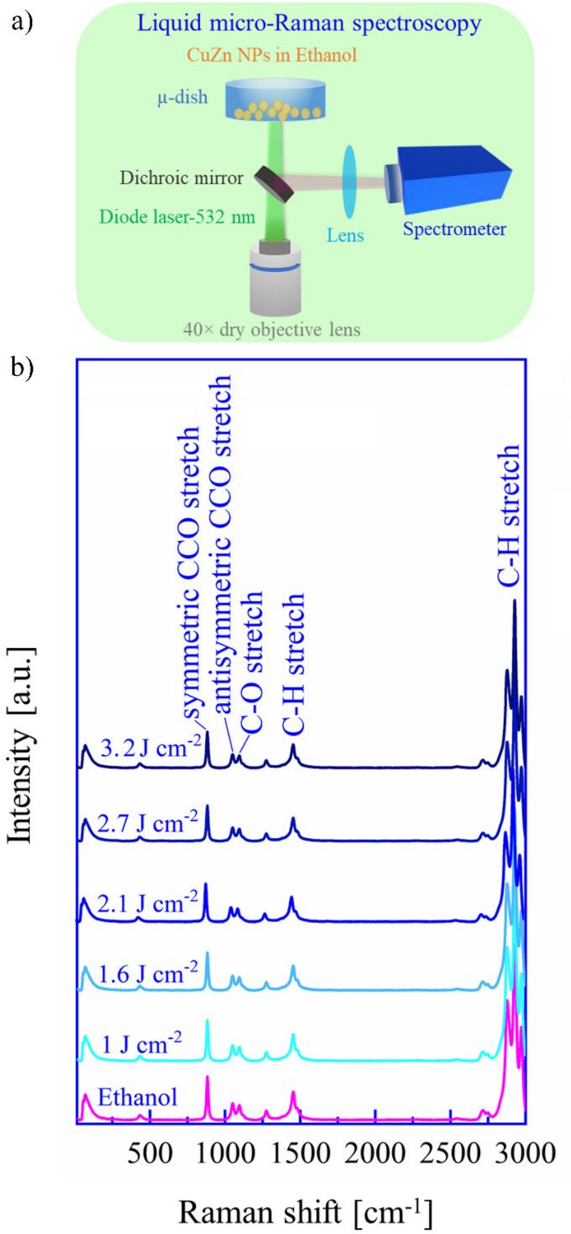


**Figure S8**. (a) Schematic of micro-Raman spectroscopy and (b) micro-Raman spectra of colloidal CuZn NPs synthesized at various *F*.

## 4.2 DLS

**Table T5.** Summary of the measured hydrodynamic properties of colloidal CuZn nanoparticles synthesized by fs-PLAL.

| $F$ [Jcm$^{-2}$] | $R_s$ [nm] | ($D_{trans}$) [µm$^2$ s$^{-1}$] | ($D_{rot}$) [s$^{-1}$] | $f_{trans}$ [kg s$^{-1}$] | $f_{rot}$ [kg m$^2$ s$^{-1}$] |
|---|---|---|---|---|---|
| 1.0 | 72 | 2.59 | 374 | $1.45 \times 10^{-9}$ | $1.00 \times 10^{-23}$ |
| 1.6 | 74 | 2.52 | 344 | $1.49 \times 10^{-9}$ | $1.09 \times 10^{-23}$ |
| 2.1 | 81 | 2.30 | 262 | $1.63 \times 10^{-9}$ | $1.43 \times 10^{-23}$ |
| 2.7 | 87 | 2.14 | 212 | $1.76 \times 10^{-9}$ | $1.77 \times 10^{-23}$ |
| 3.2 | 98 | 1.90 | 148 | $1.98 \times 10^{-9}$ | $2.53 \times 10^{-23}$ |

## 4.3 SAED/EDX/STEM/TEM

**Table T6.** Phase and reference lattice distances of Cu and CuZn alloys from ICDD database.

| | |
|---|---|
| Indexed | 01-088-3992 |
| Material | Cu |
| Crystal system | Cubic |
| Space group | Fm-3m |
| Space group number | 225 |

| No. | h | k | l | d [nm] |
|---|---|---|---|---|
| 1 | 1 | 1 | 1 | 0.20751 |
| 2 | 2 | 0 | 0 | 0.1797 |
| 3 | 2 | 2 | 0 | 0.1270 |
| 4 | 3 | 1 | 1 | 0.1083 |
| 5 | 2 | 2 | 2 | 0.1037 |
| 6 | 4 | 0 | 0 | 0.0898 |
| 7 | 3 | 3 | 1 | 0.0824 |
| 8 | 4 | 2 | 0 | 0.0803 |

| | | No. | h | k | l | d [nm] |
|---|---|---|---|---|---|---|
| Indexed | 04-006-2621 | | | | | |
| Material | $Cu_{0.70}Zn_{0.30}$ | 1 | 1 | 1 | 1 | 0.2126 |
| Crystal system | Cubic | 2 | 2 | 0 | 0 | 0.1841 |
| Space group | Fm-3m | 3 | 2 | 2 | 0 | 0.1301 |
| Space group number | 225 | 4 | 3 | 1 | 1 | 0.1110 |
| | | 5 | 2 | 2 | 2 | 0.1063 |
| | | 6 | 4 | 0 | 0 | 0.0920 |
| | | 7 | 3 | 3 | 1 | 0.0844 |
| | | 8 | 4 | 2 | 0 | 0.0823 |

| | | No. | h | k | l | d [nm] |
|---|---|---|---|---|---|---|
| Indexed | 04-004-6749 | | | | | |
| Material | $Cu_{0.75}Zn_{0.25}$ | 1 | 1 | 1 | 1 | 0.2117 |
| Crystal system | Cubic | 2 | 2 | 0 | 0 | 0.1833 |
| Space group | Fm-3m | 3 | 2 | 2 | 0 | 0.1296 |
| Space group number | 225 | 4 | 3 | 1 | 1 | 0.1105 |
| | | 5 | 2 | 2 | 2 | 0.1058 |
| | | 6 | 4 | 0 | 0 | 0.0916 |
| | | 7 | 3 | 3 | 1 | 0.0841 |
| | | 8 | 4 | 2 | 0 | 0.0819 |

| | | No. | h | k | l | d [nm] |
|---|---|---|---|---|---|---|
| Indexed | 04-004-6749 | | | | | |
| Material | $Cu_{0.85}Zn_{0.15}$ | 1 | 1 | 1 | 1 | 0.2106 |
| Crystal system | Cubic | 2 | 2 | 0 | 0 | 0.1824 |
| Space group | Fm-3m | 3 | 2 | 2 | 0 | 0.1290 |
| Space group number | 225 | 4 | 3 | 1 | 1 | 0.1100 |
| | | 5 | 2 | 2 | 2 | 0.1053 |
| | | 6 | 4 | 0 | 0 | 0.0912 |
| | | 7 | 3 | 3 | 1 | 0.0837 |
| | | 8 | 4 | 2 | 0 | 0.0815 |

| | | No. | h | k | l | d [nm] |
|---|---|---|---|---|---|---|
| Indexed | 04-003-7053 | | | | | |

| | | | | | | |
|---|---|---|---|---|---|---|
| Material | $Cu_{0.95}Zn_{0.05}$ | 1 | 1 | 1 | 1 | 0.2089 |
| Crystal system | Cubic | 2 | 2 | 0 | 0 | 0.1809 |
| Space group | Fm-3m | 3 | 2 | 2 | 0 | 0.1279 |
| Space group number | 225 | 4 | 3 | 1 | 1 | 0.1091 |
| | | 5 | 2 | 2 | 2 | 0.1044 |
| | | 6 | 4 | 0 | 0 | 0.0904 |
| | | 7 | 3 | 3 | 1 | 0.0830 |
| | | 8 | 4 | 2 | 0 | 0.0809 |

**Table T7.** SAED analysis of CuZn NPs femtosecond produced in ethanol (*F:* 1.6 $Jcm^{-2}$).

| | | **No.** | **h** | **k** | **l** | **$d_{experimental}$ [nm]** |
|---|---|---|---|---|---|---|
| Material | CuZn | 1 | 1 | 1 | 1 | 0.211 |
| Crystal system | Cubic | 2 | 2 | 0 | 0 | 0.183 |
| Space group | Fm-3m | 3 | 2 | 2 | 0 | 0.130 |
| Space group number | 225 | 4 | 3 | 1 | 1 | 0.110 |
| | | 5 | 2 | 2 | 2 | 0.105 |

**Table T8.** EDX mapping summary of CuZn NPs femtosecond produced (*F:* 1.6 $Jcm^{-2}$) in ethanol.

| Element | wt.% | at.% | |
|---|---|---|---|
| C k | 47.5 | 77.7 | |
| O k | 6.1 | 7.5 | |
| Si K | 1.1 | 0.8 | |
| Cu K | 41.8 | 12.9 | Cu:Zn |
| Zn K | 3.5 | 1.1 | 92:8 |

**Table T9.** SAED analysis of CuZn NPs femtosecond produced in ethanol (*F:* 2.1 $Jcm^{-2}$).

| | |
|---|---|
| Material | CuZn |
| Crystal system | Cubic |
| Space group | Fm-3m |
| Space group number | 225 |

| No. | h | k | l | $d_{experimental}$ [nm] |
|---|---|---|---|---|
| 1 | 1 | 1 | 1 | 0.212 |
| 2 | 2 | 0 | 0 | 0.183 |
| 3 | 2 | 2 | 0 | 0.129 |
| 4 | 3 | 1 | 1 | 0.110 |
| 5 | 3 | 3 | 1 | 0.084 |
| 6 | 4 | 2 | 0 | 0.081 |

**Table T10.** EDX mapping summary of CuZn NPs femtosecond produced at *F:* 2.1 $Jcm^{-2}$ in ethanol.

| Element | wt.% | at.% | |
|---|---|---|---|
| C k | 4 | 11.6 | |
| O k | 18.2 | 41.4 | |
| Si K | 3.5 | 4.6 | |
| Cu K | 63.8 | 36.5 | CuZn |
| Zn K | 10.5 | 5.9 | 86:14 |

**Table T11.** SAED analysis of CuZn NPs femtosecond produced in ethanol (*F:* 2.7 Jcm$^{-2}$).

| | |
|---|---|
| Material | CuZn |
| Crystal system | Cubic |
| Space group | Fm-3m |
| Space group number | 225 |

| **No.** | **h** | **k** | **l** | **$d_{experimental}$ [nm]** |
|---|---|---|---|---|
| 1 | 1 | 1 | 1 | 0.210 |
| 2 | 2 | 2 | 0 | 0.130 |
| 3 | 3 | 1 | 1 | 0.110 |
| 4 | 3 | 3 | 1 | 0.085 |
| 5 | 4 | 2 | 0 | 0.082 |

**Table T12.** EDX mapping summary of CuZn NPs femtosecond produced at *F:* 2.7 Jcm$^{-2}$ in ethanol.

| **Element** | **wt.%** | **at.%** | |
|---|---|---|---|
| C k | 27.3 | 64.0 | |
| O k | 3 | 5.2 | |
| Si K | 0.3 | 0.3 | |
| Cu K | 52.3 | 23.1 | CuZn |
| Zn K | 17.1 | 7.4 | 75:25 |

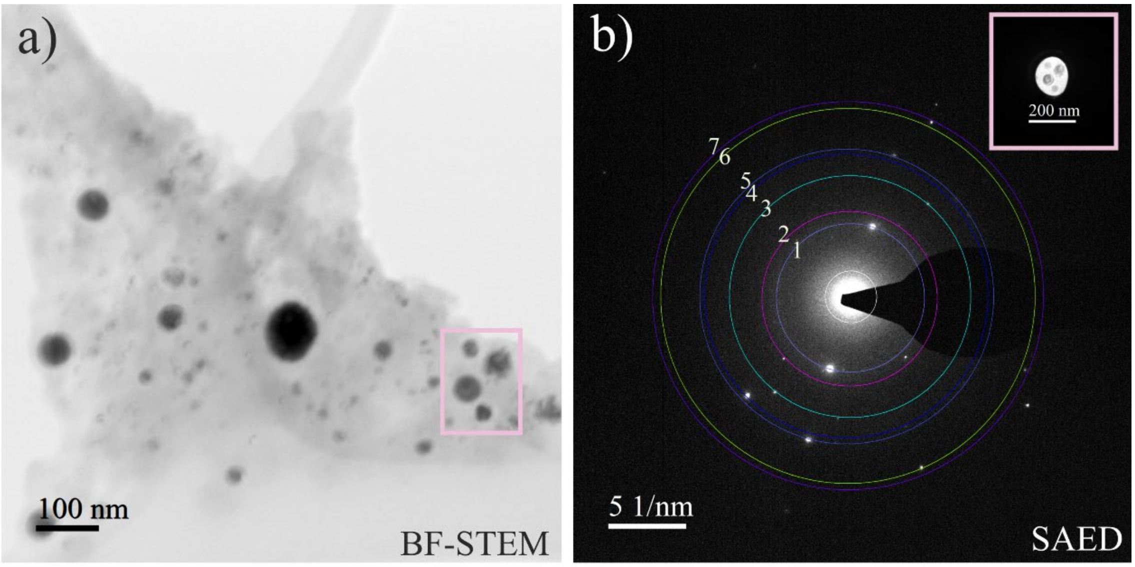


**Figure S9**. Electron microscopy analysis of CuZn nanoparticles synthesized by femtosecond laser irradiation at *F:* 2.7 Jcm$^{-2}$ in ethanol. (a) STEM; (b) SAED with aperture (inset) showing the area of interest.

**Table T13.** SAED analysis of CuZn NPs femtosecond produced in ethanol (*F:* 2.7 $Jcm^{-2}$).

| | | **No.** | **h** | **k** | **l** | **$d_{experimental}$ [nm]** |
|---|---|---|---|---|---|---|
| Material | CuZn | 1 | 1 | 1 | 1 | 0.211 |
| Crystal system | Cubic | 2 | 2 | 0 | 0 | 0.183 |
| Space group | Fm-3m | 3 | 2 | 2 | 0 | 0.129 |
| Space group number | 225 | 4 | 3 | 1 | 1 | 0.110 |
| | | 5 | 2 | 2 | 2 | 0.106 |
| | | 6 | 3 | 3 | 1 | 0.083 |
| | | 7 | 4 | 2 | 0 | 0.080 |

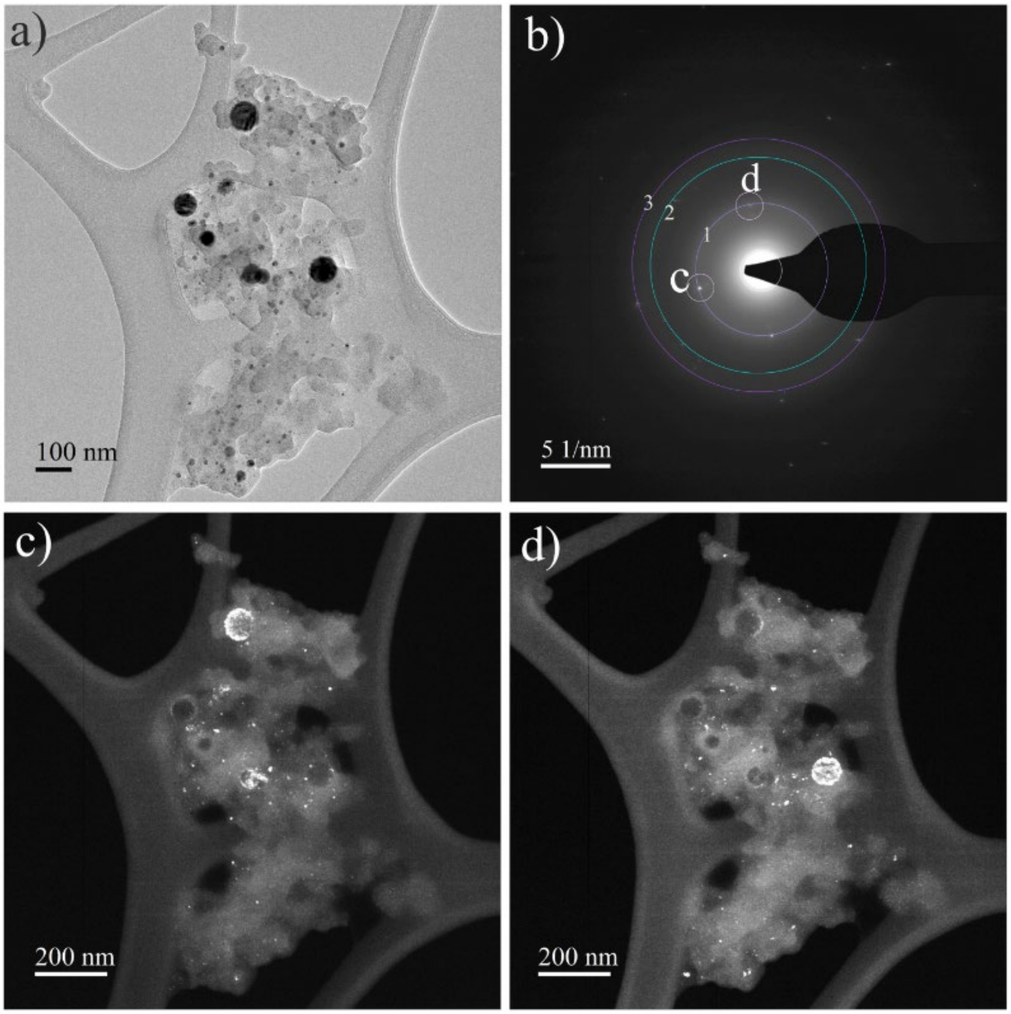


**Figure S10**. Electron microscopy studies of CuZn nanoparticles synthesized by fs-PLAL at *F:* 2.7 $Jcm^{-2}$. (a) BF-TEM; (b) SAED with specified diffraction spots on the first ring; (c) and (d) DF-TEM images from specified diffracted spots corresponding to the $1^{st}$ ring.

**Table T14.** SAED analysis of CuZn NPs femtosecond produced at *F:* 2.7 $Jcm^{-2}$.

| | | No. | h | k | l | $d_{experimental}$ [nm] |
|---|---|---|---|---|---|---|
| Indexed | 04-006-2621 | | | | | |
| Material | $Cu_{0.70}Zn_{0.30}$ | 1 | 1 | 1 | 1 | 0.210 |
| Crystal system | Cubic | 2 | 2 | 2 | 0 | 0.129 |
| Space group | Fm-3m | 3 | 3 | 1 | 1 | 0.110 |
| Space group number | 225 | | | | | |

**Table T15.** SAED analysis of CuZn NPs femtosecond produced at *F:* 3.2 $Jcm^{-2}$.

| | | No. | h | k | l | $d_{experimental}$ [nm] |
|---|---|---|---|---|---|---|
| Material | CuZn | 1 | 1 | 1 | 1 | 0.211 |
| Crystal system | Cubic | 2 | 2 | 0 | 0 | 0.183 |
| Space group | Fm-3m | 3 | 2 | 2 | 0 | 0.129 |
| Space group number | 225 | 4 | 3 | 3 | 1 | 0.111 |
| | | 5 | 4 | 0 | 0 | 0.092 |

**Table T16.** EDX mapping summary of CuZn NPs femtosecond produced at *F:* 3.2 $Jcm^{-2}$.

| Element | wt.% | at.% | |
|---|---|---|---|
| C k | 6 | 16.9 | |
| O k | 21.6 | 44.9 | |
| Si K | 0.9 | 1.0 | |
| Cu K | 60.8 | 31.8 | CuZn |
| Zn K | 10.7 | 5.4 | 85:15 |

## 4.4 Particle size distribution analysis

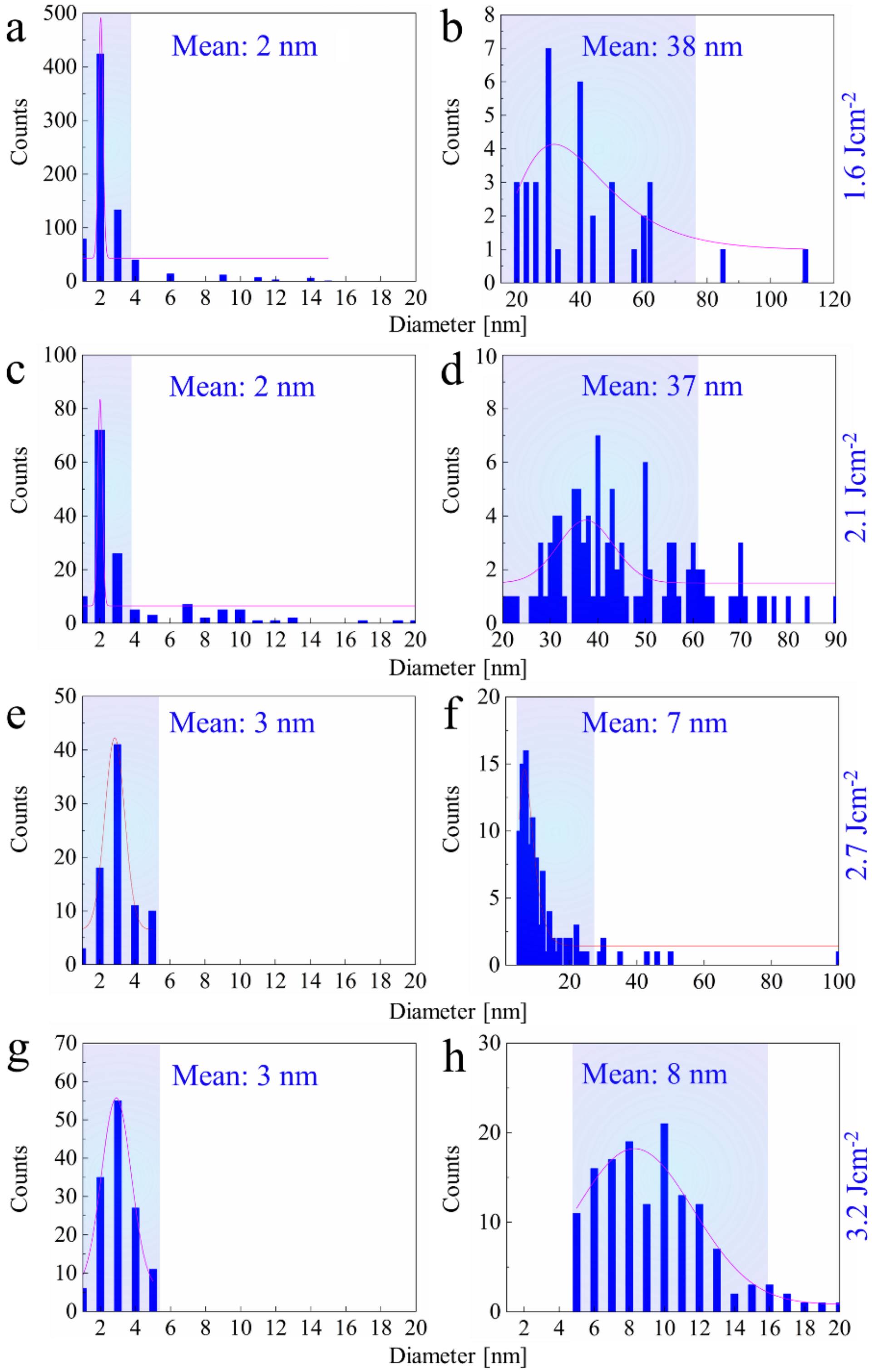


**Figure S11.** Number weighted size distribution of CuZn nanoparticles synthesized by fs-PLAL at fluences of (a,b) 1.6 $Jcm^{-2}$; (c,d) 2.1 $Jcm^{-2}$; (e,f) 2.7 $Jcm^{-2}$ and (g,h) 3.2 $Jcm^{-2}$ for the first and second mode.

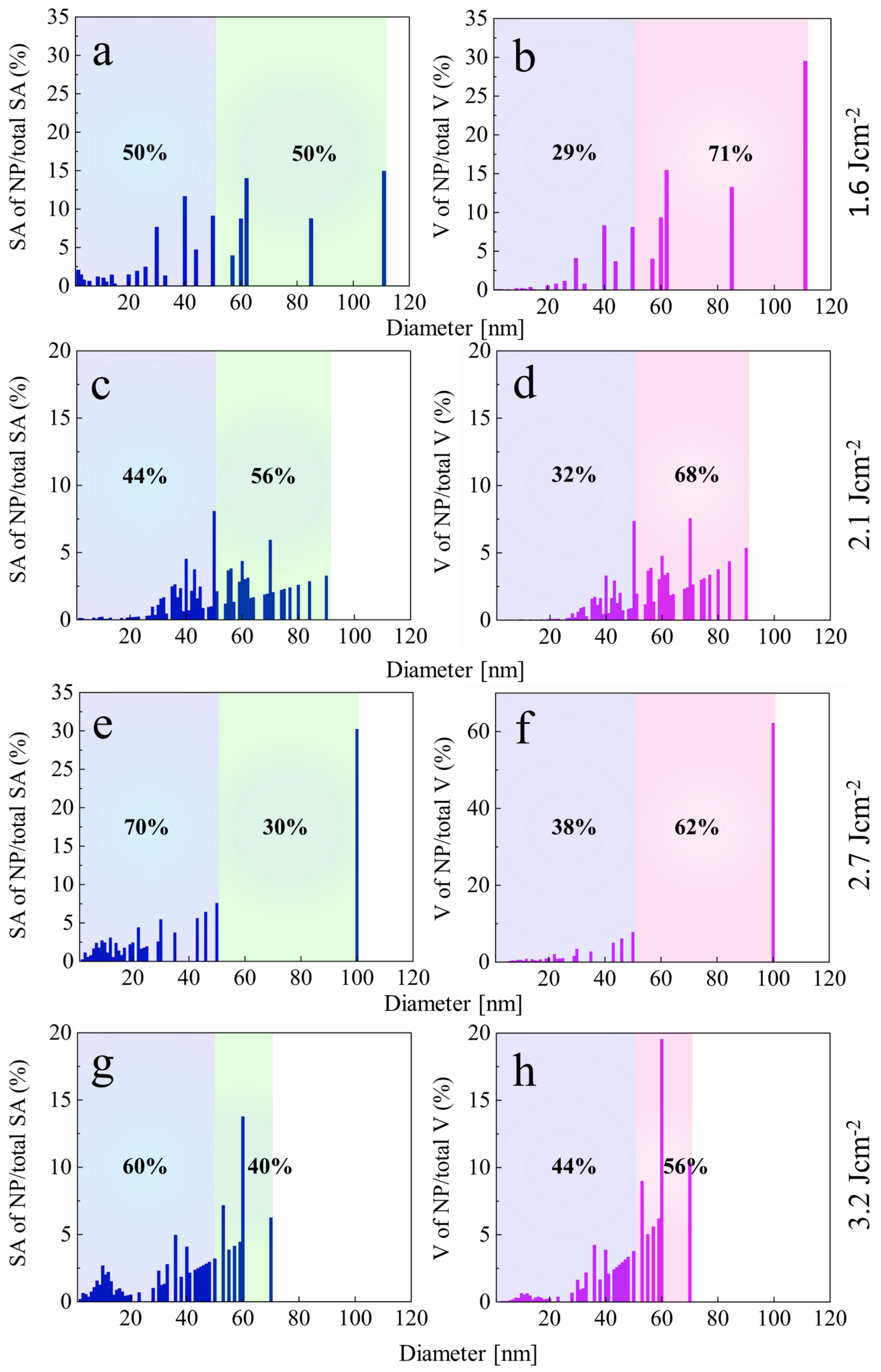


**Figure S12**. Surface and volume distribution of CuZn nanoparticles synthesized by fs-PLAL at fluences of (a,b) 1.6 $Jcm^{-2}$; (c,d) 2.1 $Jcm^{-2}$; (e,f) 2.7 $Jcm^{-2}$ and (g,h) 3.2 $Jcm^{-2}$. (a, c, e and g) surface contribution of each NP size to the total CuZn surface area; (b, d, f and h) volume contribution of each NP size to the total CuZn volume.

**Table T17.** Results for number-weighted size distribution of CuZn NPs synthesized by fs-PLAL at *F:* 1.6 $Jcm^{-2}$.

| Diameter [nm] | Counts | $SA/SA_{total}$% | SUM | $V/V_{total}$% | SUM |
|---|---|---|---|---|---|
| 1 | 79 | 0.09 | | 0.002 | |
| 2 | 424 | 2.05 | | 0.073 | |
| 3 | 133 | 1.45 | | 0.077 | |
| 4 | 40 | 0.77 | | 0.055 | |
| 6 | 14 | 0.61 | | 0.065 | |
| 9 | 12 | 1.17 | | 0.189 | |
| 11 | 7 | 1.02 | | 0.201 | |
| 12 | 3 | 0.52 | | 0.112 | |
| 14 | 6 | 1.42 | | 0.355 | |
| 15 | 1 | 0.27 | | 0.073 | |
| 20 | 3 | 1.45 | | 0.518 | |
| 23 | 3 | 1.92 | | 0.787 | |
| 26 | 3 | 2.45 | | 1.137 | |
| 30 | 7 | 7.63 | | 4.076 | |
| 33 | 1 | 1.32 | | 0.775 | |
| 40 | 6 | 11.64 | | 8.281 | |
| 44 | 2 | 4.69 | | 3.674 | |
| 50 | 3 | 9.09 | 50 | 8.087 | 29 |
| 57 | 1 | 3.94 | | 3.994 | |
| 60 | 2 | 8.73 | | 9.316 | |
| 62 | 3 | 13.98 | | 15.418 | |
| 85 | 1 | 8.76 | | 13.243 | |
| 111 | 1 | 14.94 | 50 | 29.493 | 71 |
| | 755 | | 100 | | 100 |

**Table T18.** Results for number-weighted size distribution of CuZn NPs synthesized by fs-PLAL at *F:* 2.1 $Jcm^{-2}$.

| Diameter [nm] | Counts | $SA/SA_{total}$% | SUM | V/Vtotal% | SUM |
|---|---|---|---|---|---|
| 1 | 10 | 0.004 | | 0.000 | |
| 2 | 72 | 0.116 | | 0.004 | |
| 3 | 26 | 0.094 | | 0.005 | |
| 4 | 5 | 0.032 | | 0.002 | |
| 5 | 3 | 0.030 | | 0.003 | |
| 7 | 7 | 0.138 | | 0.018 | |
| 8 | 2 | 0.051 | | 0.008 | |
| 9 | 5 | 0.163 | | 0.027 | |
| 10 | 5 | 0.201 | | 0.037 | |
| 11 | 1 | 0.048 | | 0.010 | |
| 12 | 1 | 0.058 | | 0.013 | |
| 13 | 2 | 0.136 | | 0.032 | |
| 17 | 1 | 0.116 | | 0.036 | |
| 19 | 1 | 0.145 | | 0.050 | |
| 20 | 1 | 0.161 | | 0.059 | |
| 21 | 1 | 0.177 | | 0.068 | |
| 22 | 1 | 0.194 | | 0.078 | |
| 23 | 1 | 0.213 | | 0.089 | |
| 26 | 1 | 0.272 | | 0.129 | |
| 27 | 1 | 0.293 | | 0.144 | |
| 28 | 3 | 0.947 | | 0.483 | |
| 29 | 1 | 0.338 | | 0.179 | |
| 30 | 3 | 1.087 | | 0.594 | |
| 31 | 4 | 1.548 | | 0.874 | |
| 32 | 4 | 1.650 | | 0.961 | |
| 33 | 1 | 0.438 | | 0.264 | |
| 35 | 5 | 2.467 | | 1.572 | |
| 36 | 5 | 2.610 | | 1.711 | |
| 37 | 3 | 1.654 | | 1.114 | |
| 38 | 4 | 2.327 | | 1.610 | |
| 39 | 1 | 0.612 | | 0.435 | |
| 40 | 7 | 4.512 | | 3.286 | |
| 41 | 1 | 0.677 | | 0.505 | |
| 42 | 3 | 2.132 | | 1.630 | |
| 43 | 5 | 3.724 | | 2.916 | |
| 44 | 2 | 1.559 | | 1.249 | |
| 45 | 3 | 2.447 | | 2.005 | |

| | | | | | |
|---|---|---|---|---|---|
| 46 | 1 | 0.852 | | 0.714 | |
| 48 | 1 | 0.928 | | 0.811 | |
| 49 | 1 | 0.967 | | 0.863 | |
| 50 | 8 | 8.057 | 44 | 7.334 | 32 |
| 51 | 2 | 2.095 | | 1.946 | |
| 54 | 1 | 1.174 | | 1.155 | |
| 55 | 3 | 3.656 | | 3.661 | |
| 56 | 3 | 3.790 | | 3.864 | |
| 57 | 1 | 1.308 | | 1.358 | |
| 59 | 2 | 2.804 | | 3.012 | |
| 60 | 3 | 4.351 | | 4.752 | |
| 61 | 2 | 2.998 | | 3.329 | |
| 62 | 2 | 3.097 | | 3.496 | |
| 63 | 1 | 1.599 | | 1.834 | |
| 64 | 1 | 1.650 | | 1.923 | |
| 68 | 1 | 1.862 | | 2.306 | |
| 69 | 1 | 1.918 | | 2.409 | |
| 70 | 3 | 5.922 | | 7.547 | |
| 71 | 1 | 2.030 | | 2.625 | |
| 74 | 1 | 2.206 | | 2.972 | |
| 75 | 1 | 2.266 | | 3.094 | |
| 77 | 1 | 2.388 | | 3.348 | |
| 80 | 1 | 2.578 | | 3.755 | |
| 84 | 1 | 2.842 | | 4.347 | |
| 90 | 1 | 3.263 | 56 | 5.346 | 68 |
| | 246 | | 100 | | 100 |

**Table T19.** Results for number-weighted size distribution of CuZn NPs synthesized by fs-PLAL at *F:* 2.7 $Jcm^{-2}$.

| Diameter [nm] | Counts | $SA/SA_{total}$% | SUM | V/Vtotal% | SUM |
|---|---|---|---|---|---|
| 1 | 3 | 0.009 | | 0.0002 | |
| 2 | 18 | 0.217 | | 0.0090 | |
| 3 | 41 | 1.114 | | 0.0688 | |
| 4 | 11 | 0.531 | | 0.0438 | |
| 5 | 10 | 0.755 | | 0.0777 | |
| 6 | 15 | 1.631 | | 0.2014 | |
| 7 | 16 | 2.368 | | 0.3411 | |
| 8 | 9 | 1.739 | | 0.2864 | |
| 9 | 11 | 2.691 | | 0.4984 | |
| 10 | 8 | 2.416 | | 0.4972 | |
| 11 | 3 | 1.096 | | 0.2482 | |
| 12 | 7 | 3.044 | | 0.7518 | |
| 13 | 1 | 0.510 | | 0.1366 | |
| 14 | 4 | 2.368 | | 0.6822 | |
| 15 | 2 | 1.359 | | 0.4195 | |
| 16 | 1 | 0.773 | | 0.2546 | |
| 17 | 2 | 1.746 | | 0.6107 | |
| 19 | 2 | 2.181 | | 0.8526 | |
| 20 | 2 | 2.416 | | 0.9945 | |
| 22 | 3 | 4.3861 | | 1.9855 | |
| 23 | 1 | 1.597 | | 0.7562 | |
| 24 | 1 | 1.739 | | 0.8592 | |
| 25 | 1 | 1.887 | | 0.9712 | |
| 29 | 1 | 2.540 | | 1.5159 | |
| 30 | 2 | 5.437 | | 3.3564 | |
| 35 | 1 | 3.700 | | 2.6649 | |
| 43 | 1 | 5.585 | | 4.9418 | |
| 46 | 1 | 6.391 | | 6.0499 | |
| 50 | 1 | 7.551 | 70 | 7.7694 | 38 |
| 100 | 1 | 30.207 | 30 | 0.0002 | 62 |
| | 180 | | 100 | | 100 |

**Table T20.** Results for number-weighted size distribution of CuZn NPs synthesized by fs-PLAL at *F:* 3.2 $Jcm^{-2}$.

| Diameter [nm] | Counts | $SA/SA_{total}$% | SUM | V/Vtotal% | SUM |
|---|---|---|---|---|---|
| 1 | 6 | 0.007 | | 0.000 | |
| 2 | 35 | 0.178 | | 0.008 | |
| 3 | 55 | 0.630 | | 0.045 | |
| 4 | 27 | 0.550 | | 0.052 | |
| 5 | 11 | 0.350 | | 0.041 | |
| 6 | 16 | 0.733 | | 0.104 | |
| 7 | 17 | 1.060 | | 0.176 | |
| 8 | 19 | 1.548 | | 0.293 | |
| 9 | 12 | 1.237 | | 0.264 | |
| 10 | 21 | 2.674 | | 0.633 | |
| 11 | 13 | 2.003 | | 0.521 | |
| 12 | 12 | 2.200 | | 0.625 | |
| 13 | 7 | 1.506 | | 0.463 | |
| 14 | 2 | 0.499 | | 0.165 | |
| 15 | 3 | 0.859 | | 0.305 | |
| 16 | 3 | 0.977 | | 0.370 | |
| 17 | 2 | 0.736 | | 0.296 | |
| 18 | 1 | 0.412 | | 0.176 | |
| 19 | 1 | 0.459 | | 0.207 | |
| 20 | 1 | 0.509 | | 0.241 | |
| 23 | 1 | 0.673 | | 0.367 | |
| 28 | 1 | 0.998 | | 0.662 | |
| 30 | 2 | 2.292 | | 1.627 | |
| 31 | 1 | 1.223 | | 0.898 | |
| 32 | 1 | 1.303 | | 0.988 | |
| 33 | 2 | 2.773 | | 2.166 | |
| 36 | 3 | 4.951 | | 4.218 | |
| 38 | 1 | 1.838 | | 1.654 | |
| 40 | 2 | 4.074 | | 3.858 | |
| 41 | 1 | 2.140 | | 2.077 | |
| 43 | 1 | 2.354 | | 2.396 | |
| 44 | 1 | 2.465 | | 2.567 | |
| 45 | 1 | 2.578 | | 2.746 | |
| 46 | 1 | 2.694 | | 2.934 | |
| 47 | 1 | 2.813 | | 3.129 | |
| 48 | 1 | 2.933 | | 3.333 | |
| 50 | 1 | 3.183 | 60 | 3.767 | 44 |

| | | | | | |
|---|---|---|---|---|---|
| 53 | 2 | 7.154 | | 8.974 | |
| 55 | 1 | 3.852 | | 5.014 | |
| 57 | 1 | 4.137 | | 5.581 | |
| 59 | 1 | 4.432 | | 6.190 | |
| 60 | 3 | 13.753 | | 19.530 | |
| 70 | 1 | 6.239 | 40 | 10.337 | 56 |
| | 295 | | 100 | | 100 |

### 4.5 X-ray photoelectron spectroscopy

The C 1s spectra (Figure S13) of HOPG only showed a single, slight asymmetric peak (calibrated to 284.3 eV) with a low FWHM of 1.2 eV, as well as a π→π* satellite typical of graphitic carbon.

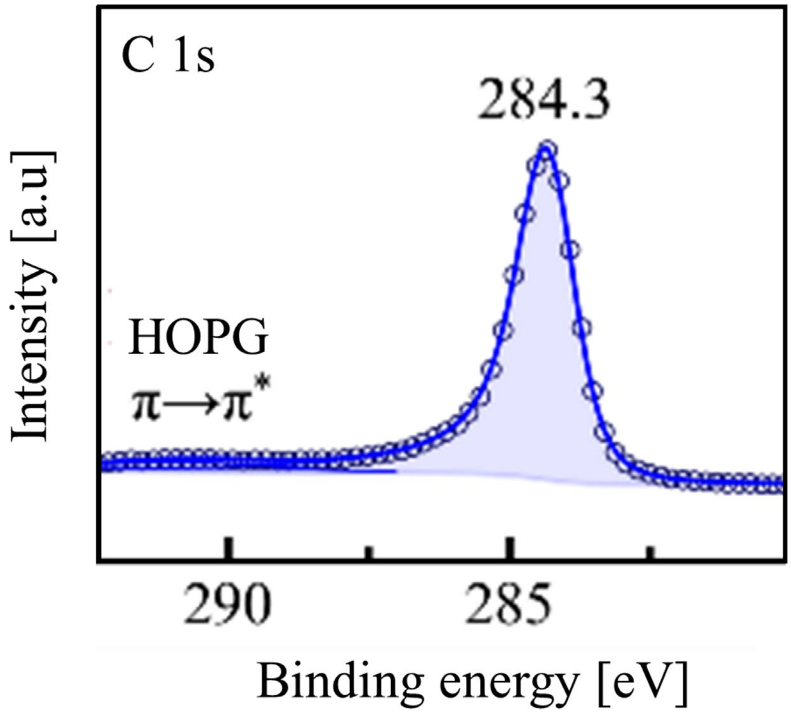


**Figure S13**. C 1s spectra of pristine HOPG. Blue solid lines are representative of the best fitting data.

## 5. Post-reaction analysis

In order to simulate the effect of reaction conditions on HOPG supported CuZn nanoparticles, NPs were also supported on carbon-coated TEM-grids and exposed to the same pretreatment and reaction conditions (120 °C, atmospheric pressure, 0.5 ml/min ethylene, 2.5 ml/min hydrogen and 7 ml/min helium). For this, a continuous-flow fixed-bed quartz reactor was used in which the samples were placed using quartz wool plugs in between each grid (Figure S14).

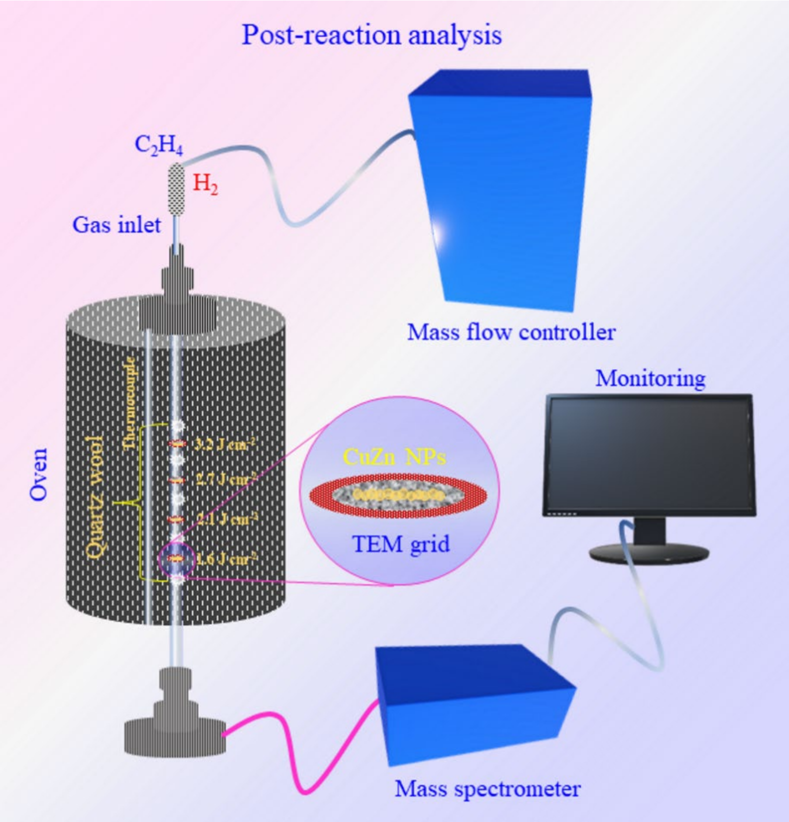


**Figure S14**. Schematic of catalytic reaction (reaction chamber and sample holder) on CuZn NPs supported on lacy carbon coated TEM grids for post-reaction analysis.

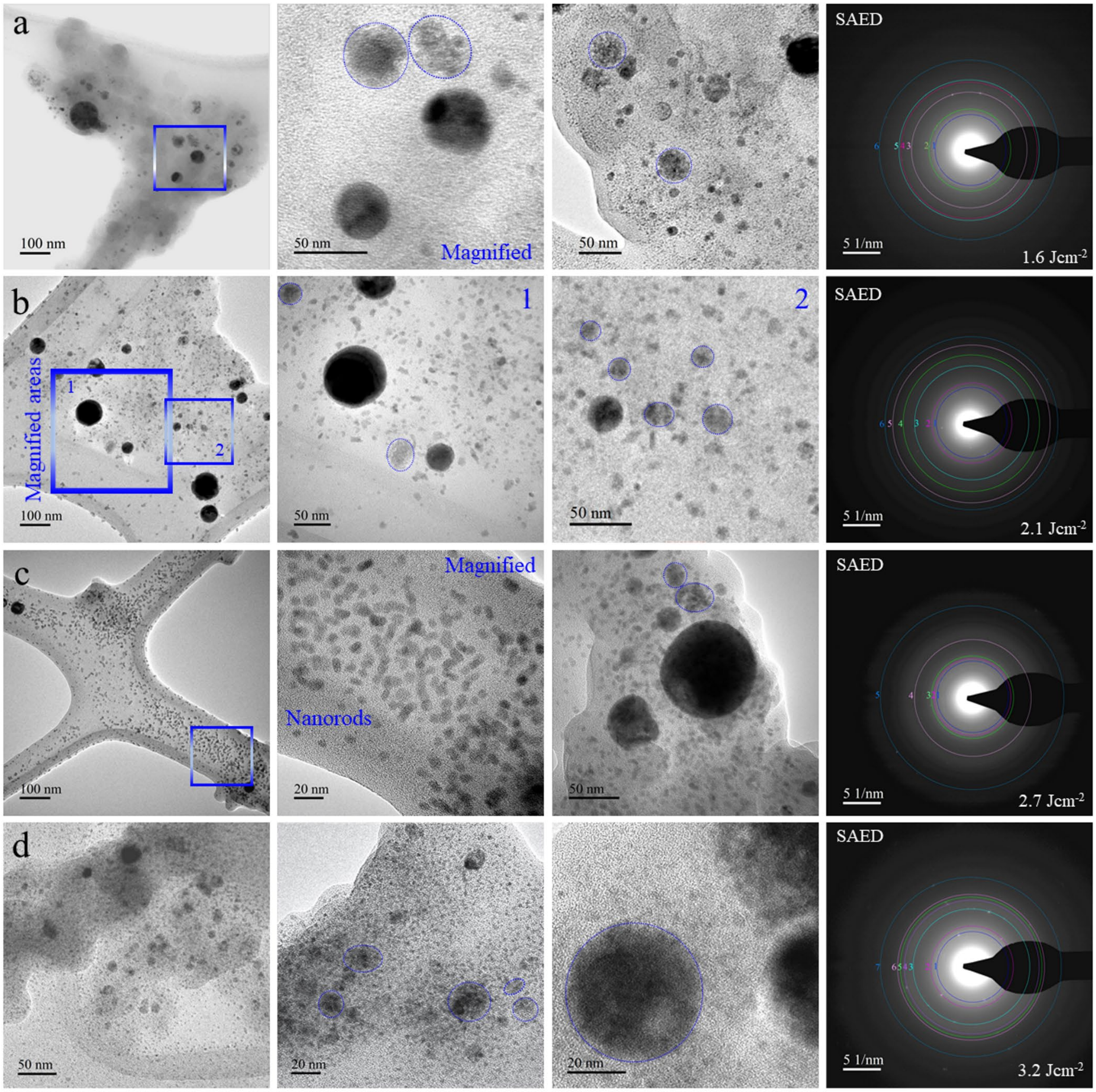


**Figure S15.** Post reaction TEM and SAED analysis of CuZn NPs synthesized by fs-PLAL at fluences of (a) 1.6 $Jcm^{-2}$; (b) 2.1 $Jcm^{-2}$; (c) 2.7 $Jcm^{-2}$; (d) 3.2 $Jcm^{-2}$. Dashed blue circles show the aggregation/agglomeration of similar sized NPs.

**Table T21.** Post-reaction SAED analysis of CuZn NPs synthesized by fs-PLAL at *F:* 1.6 Jcm$^{-2}$.

| | | No. | h | k | l | $d_{experimental}$ [nm] |
|---|---|---|---|---|---|---|
| Material | CuZn | 1 | 1 | 1 | 1 | 0.211 |
| Crystal system | Cubic | 2 | 2 | 0 | 0 | 0.183 |
| Space group | Fm-3m | 3 | 2 | 2 | 0 | 0.129 |
| Space group number | 225 | 4 | 3 | 1 | 1 | 0.110 |
| | | 5 | 2 | 2 | 2 | 0.106 |
| | | 6 | 3 | 3 | 1 | 0.083 |

**Table T22.** Post-reaction SAED analysis of CuZn NPs synthesized by fs-PLAL at *F:* 2.1 Jcm$^{-2}$.

| | | No. | h | k | l | $d_{experimental}$ [nm] |
|---|---|---|---|---|---|---|
| Material | CuZn | 1 | 1 | 1 | 1 | 0.211 |
| Crystal system | Cubic | 2 | 2 | 0 | 0 | 0.186 |
| Space group | Fm-3m | 3 | 2 | 2 | 0 | 0.131 |
| Space group number | 225 | 4 | 3 | 1 | 1 | 0.110 |
| | | 5 | 4 | 0 | 0 | 0.095 |
| | | 6 | 3 | 3 | 1 | 0.086 |

**Table T23.** Post-reaction SAED analysis of CuZn NPs synthesized by fs-PLAL at *F:* 2.7 Jcm$^{-2}$.

| | | No. | h | k | l | $d_{experimental}$ [nm] |
|---|---|---|---|---|---|---|
| Material | CuZn | 1 | 1 | 1 | 1 | 0.211 |
| Crystal system | Cubic | 2 | 2 | 0 | 0 | 0.183 |
| Space group | Fm-3m | 3 | 2 | 2 | 0 | 0.129 |
| Space group number | 225 | 4 | 4 | 2 | 0 | 0.082 |

**Table T24** Post-reaction SAED analysis of CuZn NPs synthesized by fs-PLAL at *F:* 3.2 Jcm$^{-2}$.

| | | No. | h | k | l | $d_{experimental}$ [nm] |
|---|---|---|---|---|---|---|
| Material | CuZn | 1 | 1 | 1 | 1 | 0.211 |
| Crystal system | Cubic | 2 | 2 | 0 | 0 | 0.183 |
| Space group | Fm-3m | 3 | 2 | 2 | 0 | 0.129 |
| Space group number | 225 | 4 | 3 | 1 | 1 | 0.112 |
| | | 5 | 2 | 2 | 2 | 0.106 |
| | | 6 | 3 | 3 | 1 | 0.083 |

## 6. Supplementary References


(1) Jee, Y.; Becker, M. F.; Walser, R. M. Laser-induced damage on single-crystal metal surfaces. *J. Opt. Soc. Am. B* **1988,** *5* (3), 648.

(2) Haunold, T.; Rupprechter, G. $LiO_x$-modification of Ni and $Co_3O_4$ surfaces: An XPS, LEIS and LEED study. *Surface Science* **2021,** *713*, 121915.

(3) Blume, R.; Rosenthal, D.; Tessonnier, J.-P.; Li, H.; Knop-Gericke, A.; Schlögl, R. Characterizing Graphitic Carbon with X-ray Photoelectron Spectroscopy: A Step-by-Step Approach. *ChemCatChem* **2015,** *7* (18), 2871.

(4) Doñate-Buendía, C.; Fernández-Alonso, M.; Lancis, J.; Mínguez-Vega, G. Overcoming the barrier of nanoparticle production by femtosecond laser ablation in liquids using simultaneous spatial and temporal focusing. *Photonics Research* **2019,** *7* (11), 1249.

(5) Lasemi, N.; Rentenberger, C.; Liedl, G.; Eder, D. The influence of the fluid nature on femtosecond laser ablation properties of a $SiO_2$/Si target and synthesis of ultrafine-grained Si nanoparticles. *Nanoscale Advances* **2020,** *2* (9), 3991.

(6) Lasemi, N.; Rentenberger, C.; Pospichal, R.; Cherevan, A. S.; Pfaffeneder-Kmen, M.; Liedl, G.; Eder, D. Femtosecond laser-assisted synthesis of Ni/Au BONs in various alcoholic solvents. *Applied Physics A* **2019,** *125* (8), 544.

(7) Döring, S.; Richter, S.; Nolte, S.; Tünnermann, A. In situ imaging of hole shape evolution in ultrashort pulse laser drilling. *Optics Express* **2010,** *18* (19), 20395.

(8) Lasemi, N.; Liedl, G.; Rupprechter, G. Formation of Periodic Surface Structures by Multipulse Femtosecond Laser Processing of Au-Coated Ni in Various Fluids. *ACS Applied Engineering Materials* **2023,** *1* (4), 1263.

(9) Garcell, E. M.; Singh, S. C.; Li, H.; Wang, B.; Jalil, S. A.; Guo, C. Comparative study of femtosecond laser-induced structural colorization in water and air. *Nanoscale Advances* **2020,** *2* (7), 2958.

(10) Zhang, D.; Ranjan, B.; Tanaka, T.; Sugioka, K. Carbonized Hybrid Micro/Nanostructured Metasurfaces Produced by Femtosecond Laser Ablation in Organic Solvents for Biomimetic Antireflective Surfaces. *ACS Applied Nano Materials* **2020,** *3* (2), 1855.

(11) Williamson, G. K.; Hall, W. H. X-ray line broadening from filed aluminium and wolfram. *Acta Metallurgica* **1953,** *1* (1), 22.

(12) Carbó-Argibay, E.; Bao, X.-Q.; Rodríguez-Abreu, C.; Fátima Cerqueira, M.; Petrovykh, D. Y.; Liu, L.; Kolen'ko, Y. V. Up-scaling the synthesis of $Cu_2O$ submicron particles with controlled morphologies for solar $H_2$ evolution from water. *Journal of Colloid and Interface Science* **2015,** *456*, 219.
